# CPSF1 inhibition promotes widespread use of intergenic polyadenylation sites and impairs glycolysis in prostate cancer cells

**DOI:** 10.1016/j.celrep.2024.115211

**Published:** 2025-01-22

**Authors:** Kiel T. Tietz, Braedan M. McCluskey, Conor R. Miller, Yingming Li, Sarah A. Munro, Scott M. Dehm

**Affiliations:** 1 Masonic Cancer Center, University of Minnesota, Minneapolis, MN 55455, USA; 2 Minnesota Supercomputing Institute, University of Minnesota, Minneapolis, MN 55455, USA; 3 Department of Laboratory Medicine and Pathology, University of Minnesota, Minneapolis, MN 55455, USA; 4 Lead contact

## Abstract

Localized prostate cancer can be cured by radiation or surgery, but advanced prostate cancer continues to be a clinical challenge. Altered alternative polyadenylation occurs in numerous cancers and can downregulate tumor-suppressor genes and upregulate oncogenes. We found that the cleavage and polyadenylation specificity factor (CPSF) complex factor CPSF1 is upregulated in patients with advanced prostate cancer, with high CPSF1 expression correlating with worse progression-free survival. Knockdown of CPSF1 selectively inhibited the growth of prostate cancer cells and reduced glycolytic output. Evaluating the changes in global poly(A) site usage in prostate cancer cells following CPSF1 knockdown revealed widespread usage of intergenic poly(A) sites distal to annotated 3′ UTRs, which lengthened 3′ UTRs and decreased levels of thousands of mRNAs, including key glycolysis genes. These findings uncover a role for CPSF1 in the suppression of intergenic poly(A) sites in prostate cancer and nominate CPSF1 as a therapeutic target in advanced prostate cancer.

## In brief

Tietz et al. report that CPSF1 is upregulated in patients with advanced prostate cancer and that the knockdown of CPSF1 inhibits prostate cancer growth and glycolytic output. Knockdown of CPSF1 increases the usage of thousands of poly(A) sites in intergenic sequences downstream of annotated 3′ UTRs, leading to transcript lengthening and destabilization.

## Graphical abstract

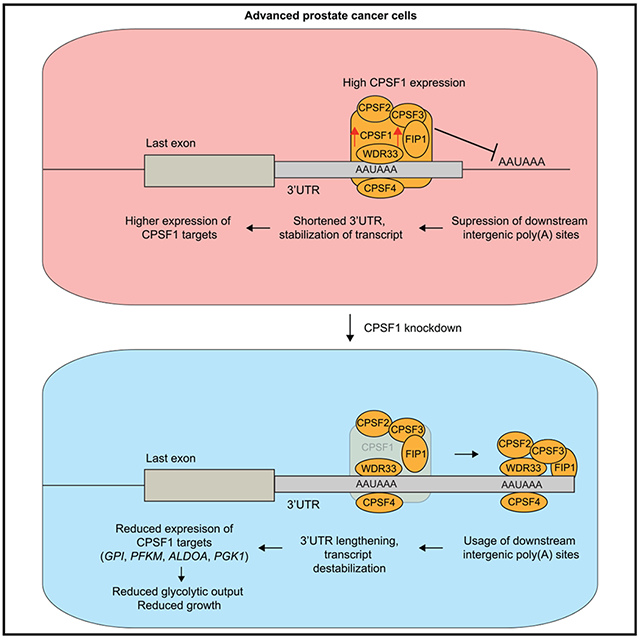

## INTRODUCTION

Prostate cancer is the second leading cause of male cancer death in the United States. While localized disease can be cured by radiation or surgery, advanced prostate cancer presents a clinical challenge. Advanced prostate cancer can initially be controlled with endocrine therapies that inhibit activity of the androgen receptor (AR), including androgen deprivation, the AR antagonist enzalutamide, and the androgen synthesis inhibitor abiraterone. However, these tumors will inevitably develop resistance in the form of castration-resistant prostate cancer (CRPC).^1^ There are currently no curative therapies for CRPC, thus demonstrating a need for the identification of alternative therapeutic targets and vulnerabilities.

mRNA transcripts undergo cleavage and polyadenylation ~25 bp downstream of the poly(A) site located within the 3′ UTR, which has the canonical sequence AAUAAA. Alternative polyadenylation (APA) is the usage of a poly(A) site that is proximal or distal to the predominant poly(A) site in the transcript and provides a mechanism to modify the 3′ end of mRNA transcripts. Tumor-specific regulation of APA, leading to the overexpression of oncogenes or the inactivation of tumor suppressors, is widespread in cancer.^2–8^ In prostate cancer, APA of *AR* mRNAs can promote the synthesis of constitutively active AR variants, including AR-variant 7 (AR-V7),^9^ and cell cycle progression of prostate cancer cells can be enhanced through Sam68/XRN2-dependent APA events.^10^

mRNA polyadenylation is initiated by four core complexes that determine which poly(A) site in a transcript will be selected for initiating endonucleolytic cleavage. These complexes include the cleavage and polyadenylation specificity factor (CPSF) complex, the cleavage stimulation factor (CstF) complex, the cleavage factor I (CFIm) complex, and the CFIIm complex.^11^ The CPSF complex factor CPSF1 has been found to regulate the growth of several cancer subtypes by altering polyadenylation and splicing.^9,12–14^ Multiple other polyadenylation factors, such as CPSF3, CPSF4, CSTF2, CPSF6, NUDT21, PCF11, and CLP1, also function in cancer progression,^15–29^ highlighting that factors involved in mRNA polyadenylation may be a therapeutic vulnerability of cancer.

While APA has become recognized as a mechanism of dysregulation in cancer, less is known about direct targets and pathways regulated by APA in cancer, specifically in prostate cancer. In this study, we found that CPSF1 mRNA expression levels are elevated in CRPC tumors relative to primary prostate cancer and that high CPSF1 expression predicted shorter progression-free survival. Knockdown of CPSF1 inhibited the growth of prostate cancer cells but not that of a benign epithelial prostate cell line. The selective growth inhibition of prostate cancer cells following CPSF1 knockdown was associated with impaired glycolysis and deregulated expression of glycolysis-regulating genes. Investigation of the mechanism underlying this selective CPSF1 dependence revealed that CPSF1 regulates global gene expression through repression of thousands of poly(A) sites, many of which are located distal to annotated 3′ UTRs. CPSF1-dependent targets included glycolytic genes, providing a mechanistic underpinning for impaired glycolysis in prostate cancer cells following CPSF1 inhibition. Collectively, this work identifies CPSF1 as a factor required for glycolytic output of prostate cancer cells.

## RESULTS

### CPSF1 is required for growth of prostate cancer cells

**Figure 1.**
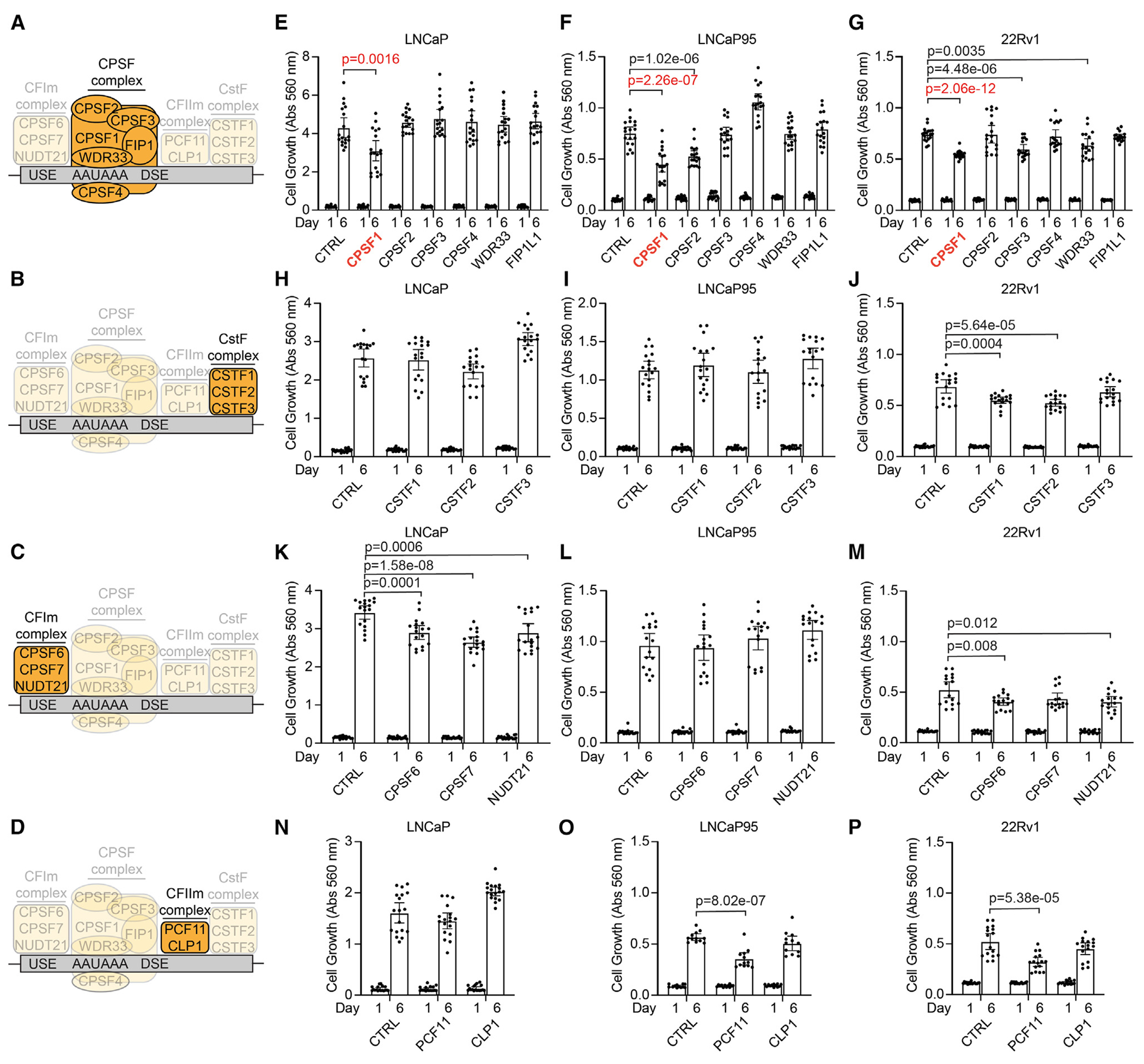
Growth of hormone-sensitive and -insensitive prostate cancer cell line models requires CPSF1 (A–D) Illustration of the CPSF, CstF, CFIm, and CFIIm complexes. (E–P) Growth of LNCaP, LNCaP95, and 22Rv1 cells transfected with indicated siRNAs targeting CPSF, CstF, CFIm, and CFIIm complex factors. A non-targeting siRNA (CTRL) was used as a control. Cell growth was measured on days 1 and 6 post-transfection using crystal violet staining. Data are mean ± 95% confidence interval (CI) of 3 independent experiments, each performed as 6 biological replicates (*n* = 18). Significance was assessed by unpaired two-sided t tests. USE, upstream sequence element; DSE, downstream sequence element; Abs, absorbance.

The CPSF, CstF, CFIm, and CFIIm complexes recognize and bind the poly(A) site and flanking sequences to promote endonucleolytic cleavage of mRNA transcripts (Figures 1A–1D). To determine the role of mRNA polyadenylation in prostate cancer, we used gene-specific small interfering RNAs (siRNAs) to assess the effects of knocking down each poly(A) factor on the growth of hormone-sensitive LNCaP cells and hormone-insensitive LNCaP95 and 22Rv1 cells (Figures 1, S1A, and S1B). We noted several cell line-selective effects, such as the knockdown of CPSF6 and NUDT21 inhibiting the growth of 22Rv1 and LNCaP cells (Figures 1K and 1M) and the knockdown of PCF11 inhibiting the growth of 22Rv1 and LNCaP95 cells (Figures 1O and 1P). However, only the knockdown of CPSF1 significantly reduced growth across all cell lines (Figures 1E–1G). These results identify a role for CPSF1 in supporting the growth of prostate cancer cells *in vitro*.

### High CPSF1 expression is associated with prostate cancer progression

**Figure 2.**
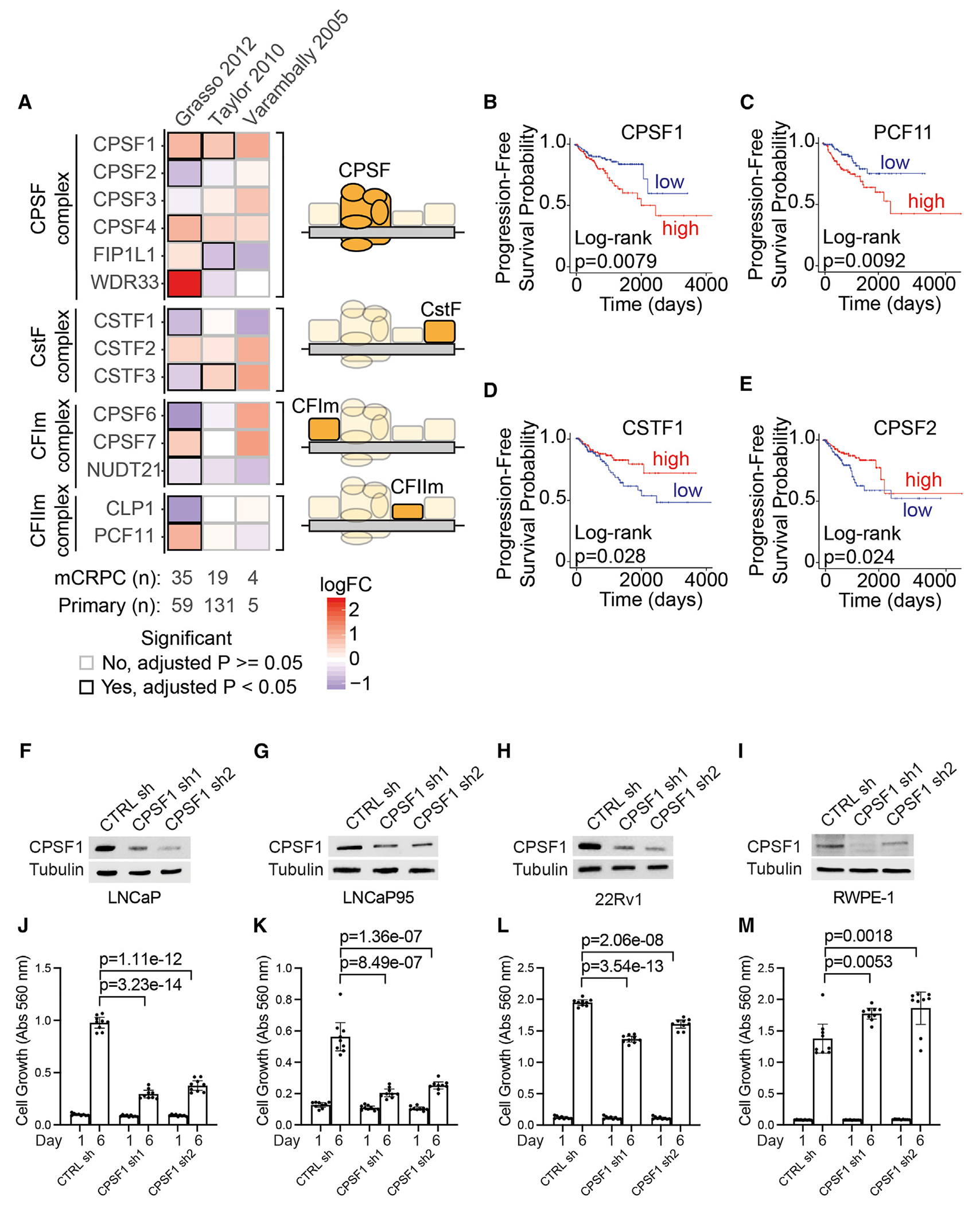
Altered expression of *CPSF1* mRNA is associated with prostate cancer progression (A) Indicated mRNAs encoding factors in the CPSF, CstF, CFIm, and CFIIm complexes were tested for differential expression between metastatic CRPC (mCRPC) and primary prostate cancer specimens in public microarray datasets. (B–E) For each indicated gene, patients in the top vs. bottom quartiles of gene expression were tested for associations with progression-free survival using TCGA RNA-seq data. (F–I) Western blot of CPSF1 (top) and Tubulin (bottom) in LNCaP (F), LNCaP95 (G), 22Rv1 (H), and RWPE-1 (I) cells infected with lentivirus encoding shRNAs targeting CPSF1 (CPSF1 sh1 and CPSF1 sh2) or a non-targeting shRNA (CTRL sh). (J–M) Growth of LNCaP, LNCaP95, 22Rv1, and RWPE-1 cells infected as in (F)–(I). Cell growth was measured on days 1 and 6 post-seeding using crystal violet staining. Data are mean ± 95% CI. Significance was assessed by unpaired two-sided t tests. Abs, absorbance.

To complement the siRNA screening of factors in the CPSF, CstF, CFIm, and CFIIm complexes, we examined three publicly available gene expression datasets wherein primary prostate cancer and metastatic CRPC tissues were analyzed.^30–32^ CPSF1, and to a lesser extent CPSF4, displayed consistent upregulation in CRPC tumors relative to primary tumors (Figure 2A). We also tested for gene expression associations with prostate cancer progression using TCGA data curated specifically for survival analysis.^33^ High expression of CPSF1 and PCF11 and low expression of CPSF2 and CSTF1 were associated with faster disease progression (Figures 2B–2E and S1C). Thus, components of the machinery regulating mRNA cleavage and polyadenylation display altered expression in prostate cancer, with high CPSF1 correlating strongly with progression to metastatic CRPC. To confirm an important role for CPSF1 in regulating the growth of prostate cancer cells and rule out off-target effects from screening with siRNA pools, we used two independent lentivirus short hairpin RNAs (shRNAs) to stably knockdown CPSF1 in LNCaP, LNCaP95, and 22Rv1 cells and the benign epithelial prostate cell line RWPE-1 (Figures 2F–2I). As expected, the stable knockdown of CPSF1 inhibited the growth of all three prostate cancer cell lines (Figures 2J–2L) but, interestingly, had no inhibitory growth effect on RWPE-1 cells (Figure 2M). These data demonstrate that increased levels of CPSF1 are associated with prostate cancer progression and that CPSF1 is required for the growth of prostate cancer cells but not benign epithelial prostate cells.

### CPSF1 regulates global gene expression and the glycolytic rate of prostate cancer cells

**Figure 3.**
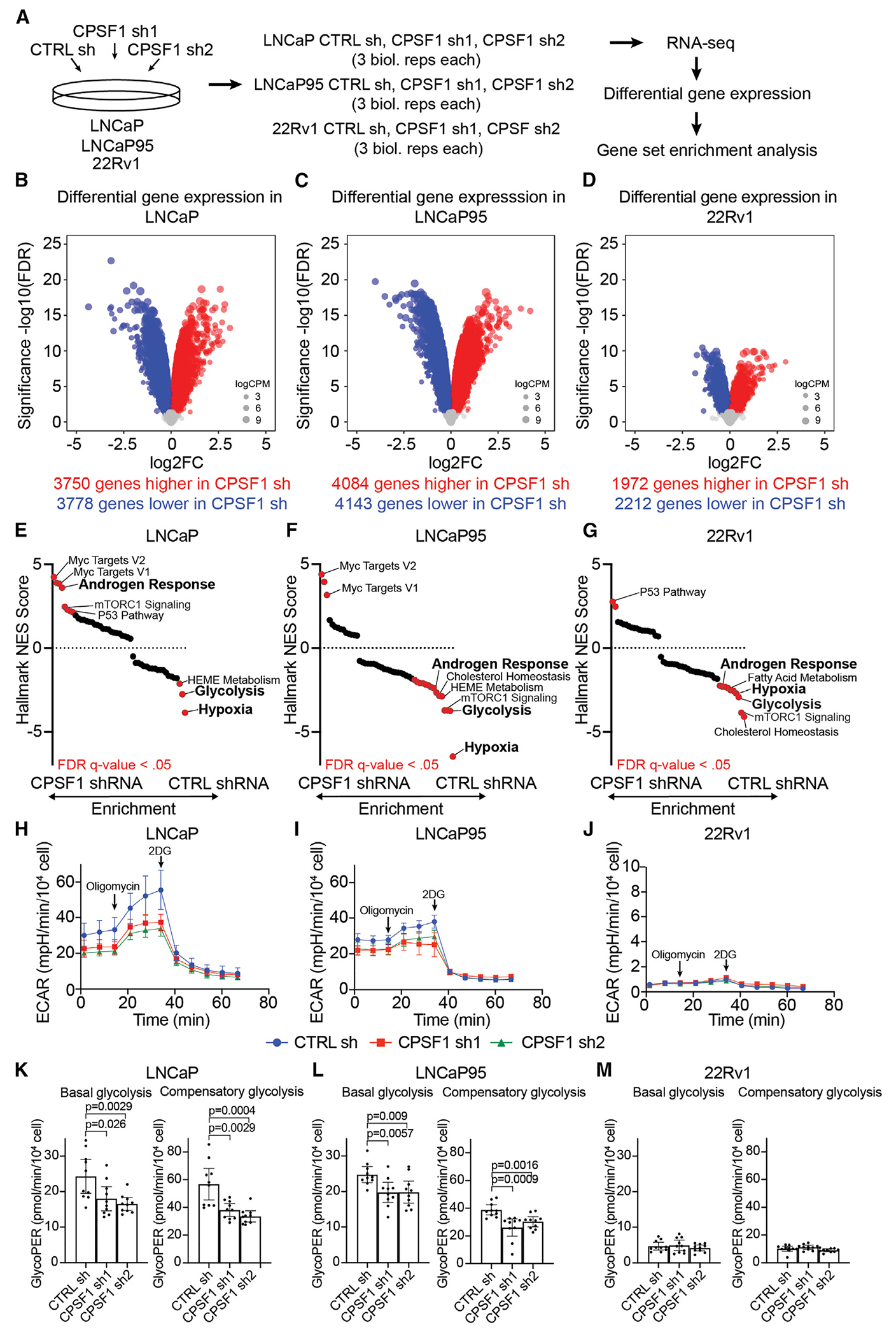
CPSF1 regulates the expression of genes encoding glycolytic factors in prostate cancer (A) RNA-seq experimental design for knockdown of CPSF1 with two independent shRNAs targeting CPSF1 (shCPSF1-1 and -2) or control shRNA (shCTRL). (B–D) Volcano plots showing global downregulation and upregulation of genes upon CPSF1 knockdown in LNCaP, LNCaP95, and 22Rv1 cells. Genes in volcano plots are colored blue or red based on whether they are downregulated or upregulated with false discovery rate (FDR) < 0.05. (E–G) Normalized enrichment scores for all 50 MSigDB Hallmark signatures derived from gene set enrichment analysis (GSEA) in LNCaP, LNCaP95, and 22Rv1 cells (differential expression in shCTRL vs. shCPSF1). Hallmark signatures are colored red if the FDR < 0.05. (H–J) ECAR output of CPSF1 shRNA- or CTRL shRNA-infected LNCaP, LNCaP95, and 22Rv1 cells. (K–M) Basal and compensatory glycolysis of CPSF1 shRNA- or CTRL shRNA-infected LNCaP, LNCaP95, and 22Rv1 cells. ECAR, extracellular acidification rate; GlycoPER, glycolytic proton efflux rate. Data are mean ± 95% CI. Significance was assessed by unpaired two-sided t tests.

To identify CPSF1-regulated pathways required for prostate cancer growth, we performed RNA sequencing (RNA-seq) on LNCaP, LNCaP95, and 22Rv1 cells with stable knockdown of CPSF1 (Figure 3A). Unexpectedly, differential gene expression revealed a similar scale of global downregulation and upregulation of genes upon CPSF1 shRNA knockdown, with thousands of genes showing altered expression and a subset of downregulated and upregulated genes conserved across each cell line (Figures 3B–3D and S1D; Table S1). Using gene set enrichment analysis (GSEA), we tested the positive or negative enrichment of gene signatures in the Molecular Signatures Database (mSigDB)^34^ (Figures 3E–3G; Table S1). Despite many individual genes being upregulated after CPSF1 knockdown, LNCaP95 and 22Rv1 cell lines displayed very few Hallmark gene signatures with positive enrichment in CPSF1 vs. control knockdown conditions. Instead, there were many Hallmark gene signatures with negative enrichment in LNCaP95 and 22Rv1 cell lines under CPSF1 vs. control knockdown conditions. LNCaP cells displayed the reciprocal trend of a high number of Hallmark gene signatures with positive enrichment under CPSF1 vs. control knockdown conditions but few Hallmark gene signatures with negative enrichment.

To understand if these effects of CPSF1 knockdown reflected broad inhibition of mRNA cleavage and polyadenylation, we compared the transcriptomic signatures of the CPSF1 knockdown with the transcriptomic signature of JTE-607, a cleavage and polyadenylation inhibitor that targets the mRNA endonuclease CPSF3.^35^ We first developed shCPSF1_UP and shCPSF1_DOWN gene sets that consisted of genes up- or down-regulated by CPSF1 knockdown in LNCaP, LNCaP95, and 22Rv1 cells (Table S2) and tested enrichment of these genes in gene expression datasets generated from HepG2 and HeLa cells treated with 1 or 10 μM JTE-607.^35^ We found that the shCPSF1_UP gene set was positively regulated by JTE-607 treatment, and the shCPSF1_DOWN gene set was negatively regulated by JTE-607 treatment (Figure S2A). Similarly, we developed JTE-607_UP and JTE-607_DOWN gene sets that consisted of genes up- or downregulated by JTE-607 treatment in HepG2 and HeLa cells (Table S2). We found that the JTE-607_DOWN gene set was negatively regulated by CPSF1 knockdown in LNCaP95 and 22Rv1 cells but not LNCaP cells (Figure S2B). The JTE-607_UP gene set was positively regulated by CPSF1 knockdown in LNCaP and 22Rv1 cells but not LNCaP95 cells (Figure S2B). Collectively, these data indicate that CPSF1-regulated genes are targets of mRNA cleavage and polyadenylation but that not all targets of mRNA cleavage and polyadenylation are regulated by CPSF1. To test this concept further, we evaluated the effects of JTE-607 treatment on the growth of LNCaP, LNCaP95, 22Rv1, and RWPE-1 cells. We found that each cell line displayed similar sensitivity to growth inhibition by JTE-607 (Figure S2C), which contrasts with the lack of growth inhibition in RWPE-1 cells stably expressing CPSF1 shRNA (Figure 2M).

To investigate CPSF1-specific regulatory effects in more detail, we explored enriched Hallmark gene signatures. Interestingly, we found negative enrichment of the HALLMARK_ANDROGEN_RESPONSE gene set following CPSF1 shRNA knockdown in LNCaP95 and 22Rv1 cells (Figures 3F and 3G) but the opposite pattern of positive enrichment of this gene set in LNCaP cells (Figure 3E). LNCaP95 and 22Rv1 cells both express AR-V7, which is a constitutively active splicing variant that requires CPSF1 binding to an APA site in intron 3 of the *AR* gene.^9^ Negative enrichment of the HALLMARK_ANDROGEN_RESPONSE gene set is consistent with this finding because many of the genes in this Hallmark are regulated transcriptionally by AR-V7.^36^ Conversely, LNCaP cells are AR-V7 negative, which suggests that CPSF1 may function to negatively regulate AR signaling in this context. Because of this finding, we tested whether CPSF1 regulates the response of prostate cancer cells to the AR antagonist enzalutamide. The stable knockdown of CPSF1 did not alter the enzalutamide-resistant growth phenotype of LNCaP95 cells, and the overexpression of a cDNA encoding CPSF1 did not reverse the enzalutamide-sensitive growth phenotype of LNCaP cells (Figures S2D–S2F). These findings indicate that CPSF1 is not a key determinant of enzalutamide responsiveness of prostate cancer cells.

Next, we explored non-androgen signaling pathways regulated by CPSF1. GSEA revealed Hallmark gene sets that displayed consistent negative enrichment in all 3 cell lines after CPSF1 shRNA knockdown, such as the HALLMARK_HYPOXIA and HALLMARK_GLYCOLYSIS gene sets (Figures 3E–3G). Because of this finding, we examined the glycolytic rates of LNCaP, LNCaP95, and 22Rv1 cells with stable CPSF1 knockdown (Figures 3H–3J). We observed that CPSF1 knockdown decreased basal glycolysis and compensatory glycolysis in LNCaP and LNCaP95 cells, whereas similar effects were not evident in 22Rv1 cells due to the very low general glycolytic output relative to LNCaP and LNCaP95 cells (Figures 3K–3M, S3A, and S3B). Inhibition of cell growth and glycolysis by CPSF1 knockdown was also apparent when experiments were performed under physiological (2 mM) levels of glucose (Figures S3C–S3I). Conversely, the overexpression of CPSF1 in LNCaP cells did not increase basal glycolysis or compensatory glycolysis compared to the GFP control (Figures S3J and S3K). Collectively, these data demonstrate that CPSF1 is necessary to maintain glycolysis in prostate cancer cells but CPSF1 overexpression is not sufficient to increase glycolytic output.

To determine if lowered glycolytic output was the basis for the cell growth inhibition by CPSF1 knockdown, we examined if the addition of various metabolites generated from glycolysis rescued the growth inhibition caused by CPSF1 shRNA knockdown. We first incubated CPSF1 shRNA-infected LNCaP95 cells with the glycolysis end product pyruvate but found no rescue of growth (Figure S3L). Because nucleotide synthesis, nicotinamide adenine dinucleotide (NAD+) metabolism, citric acid cycle, and fatty acid synthesis all require metabolites produced by glycolysis,^37^ we incubated CPSF1 shRNA-infected LNCaP95 cells with end products or intermediates from each of these pathways. However, none of these metabolites, alone or in combination, rescued the growth inhibition caused by CPSF1 knockdown (Figures S3M–S3O). These data indicate that the growth inhibition caused by CPSF1 knockdown is due not only to lower levels of metabolites sourced from glycolysis but also to the coordinate disruption of multiple additional pathways (as illustrated by GSEA; Figures 3E–3G).

### CPSF1 regulates widespread poly(A) usage outside 3′ UTR sequences

**Figure 4.**
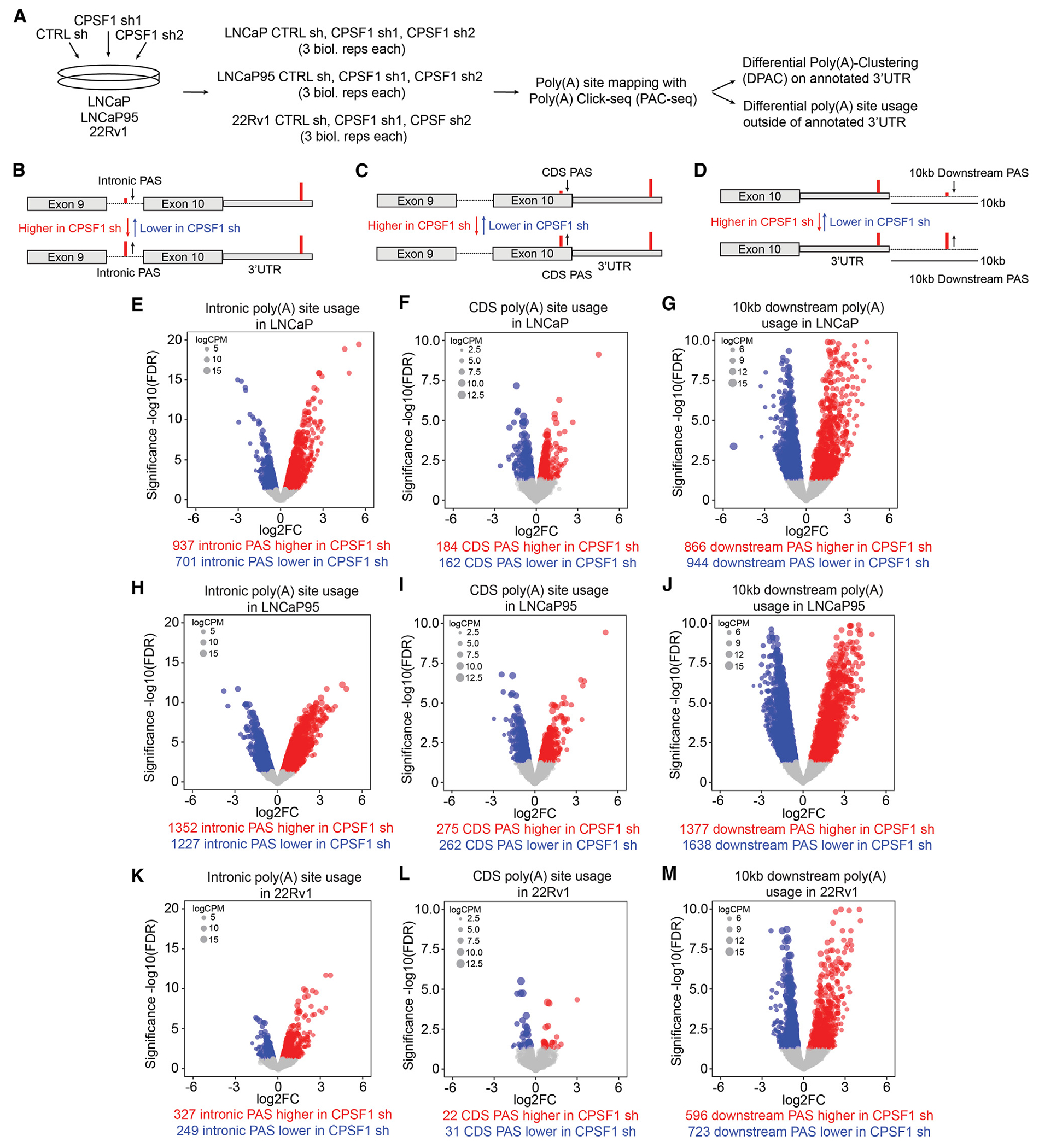
CPSF1 preferentially regulates the usage of poly(A) sites in intergenic sequences beyond annotated 3′ UTRs (A) PAC-seq experimental design for knockdown of CPSF1 with two independent shRNAs targeting CPSF1 (shCPSF1-1 and −2) or control shRNA (shCTRL). (B–D) Diagrams illustrating how changes in PAC-seq reads from CPSF1 shRNA and CTRL shRNA were quantified in intronic regions, CDS regions, or downstream of annotated 3′ UTR sequence (10 kb downstream region). (E–M) Volcano plots showing higher or lower PAS usage in intronic, CDS, or 10 kb downstream regions in CPSF1 shRNA vs. CTRL shRNA in LNCaP cells (E–G), LNCaP95 cells (H–J), and 22Rv1 cells (K–M). PASs in volcano plots are colored blue or red based on whether they are upregulated or downregulated with FDR < 0.05. PAS, poly(A) site; CDS, coding sequence.

Because CPSF1 is a core eukaryotic mRNA polyadenylation factor, we examined the role of CPSF1 in regulating global polyadenylation in prostate cancer. We used Poly(A) Click-seq (PAC-seq)^38,39^ to identify changes in global poly(A) site usage following CPSF1 knockdown in LNCaP, LNCaP95, and 22Rv1 cells (Figure 4A). We initially focused on poly(A) changes occurring within annotated 3′ UTRs by analyzing PAC-seq data with the differential poly(A) clustering (DPAC) analysis algorithm^40^ (Figure S4A). Analysis with DPAC indicated that the number of 3′ UTR-lengthening and/or -shortening events caused by CPSF1 shRNA knockdown were much lower than would be expected from the large number of genes displaying differential expression. For instance, LNCaP cells displayed 260 total 3′ UTR-lengthening/-shortening events, LNCaP95 cells displayed 794 total events, and 22Rv1 cells only displayed 61 total events (Figure S4B; Table S3).

Because DPAC analysis only quantifies changes in poly(A) site usage within annotated 3′ UTRs, and because APA has been found to occur outside of 3′ UTRs,^3,29,41^ we examined the effects of CPSF1 knockdown on poly(A) site usage outside of annotated 3′ UTRs. We partitioned the genome into four regions, (1) 3′ UTRs, (2) introns, (3) exon coding sequences (CDSs), and (4) genomic regions extending 10 kb downstream of the end of annotated 3′ UTRs (10 kb downstream sequence), and quantified PAC-seq read changes upon CPSF1 knockdown within each region (Figures 4B–4D). We limited the distance downstream of the end of annotated 3′ UTRs to 10 kb based on our finding that this cutoff captured a high number of intergenic PAC-seq reads while avoiding reads occurring in neighboring genes (Figure S4C). Examining the PAC-seq read changes in each region revealed thousands of poly(A) sites that were not considered by DPAC for calculating 3′ UTR lengthening/shortening (Figures S4D–S4L). These poly(A) sites displayed higher or lower usage following CPSF1 knockdown in all three cell lines (Figures 4E–4M; Table S3). While CDS regions displayed a small difference in poly(A) site usage upon CPSF1 knockdown, intronic and 10 kb downstream regions showed extensive differences in poly(A) site usage, with the 10 kb downstream regions displaying the largest change in poly(A) site usage across all three cell lines (Figures 4G–4J, and 4M). These data demonstrate that CPSF1 preferentially regulates the usage of poly(A) sites in intronic sequences and genomic sequences beyond annotated 3′ UTRs and has less pronounced roles in regulating poly(A) site usage in CDS regions and annotated 3′ UTRs.

### CPSF1-regulated poly(A) site usage alters gene expression

**Figure 5.**
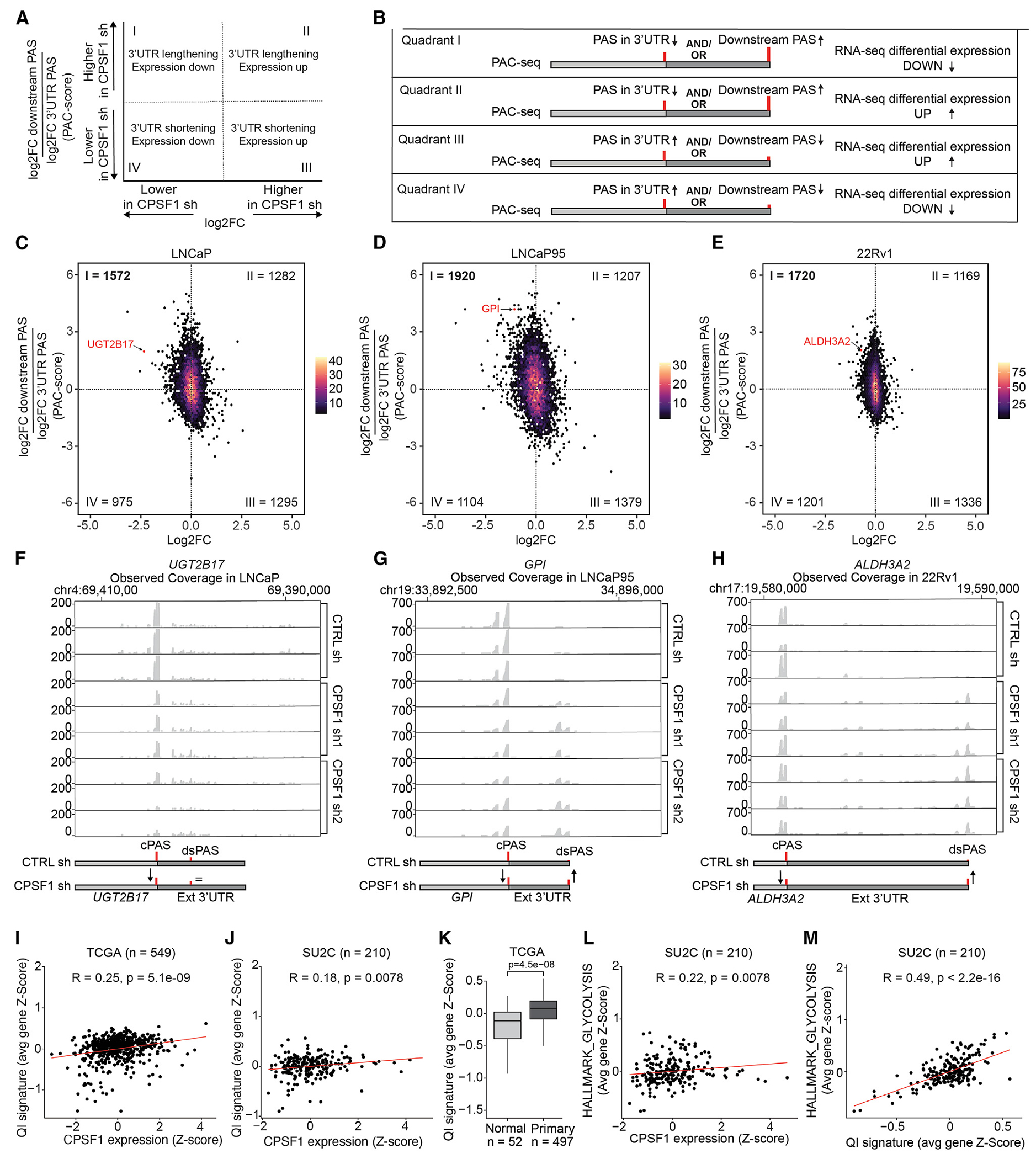
CPSF1-regulated poly(A) site usage corresponds with expression changes (A and B) Example plot and diagram illustrating the 3′ UTR length changes (PAC score) and expression changes that occur in each quadrant. (C–E) Density plots illustrating the 10 kb downstream PAC score and RNA-seq differential expression of genes in LNCaP, LNCaP95, and 22Rv1 cells infected with CPSF1 shRNA. Numbers represent the amount of genes represented in each quadrant. (F–H) Coverage of PAC-seq reads for representative quadrant I genes *UGT2B17* in LNCaP cells, *GPI* in LNCaP95 cells, and *ALDH3A2* in 22Rv1 cells. Diagrams on the bottom illustrate the effect of CPSF1 knockdown on 3′ UTR length for each indicated transcript. Red lines denote poly(A) sites called by DPAC analysis. (I and J) Pearson correlation analysis of the QI gene signature (avg gene *Z* score) to CPSF1 *Z* score in TCGA (I) and SU2C (J) patient datasets. (K) The QI gene signature (avg gene *Z* score) plotted in normal and primary prostate cancer patient data from TCGA. (L and M) Pearson correlation analysis of the HALLMARK_GLYCOLYSIS gene set (avg gene *Z* score) to CPSF1 *Z* score (L) and QI gene signature (avg gene *Z* score) (M) in the SU2C patient dataset. Significance was assessed by unpaired two-sided t tests. Q1, quadrant I; cPAS, canonical poly(A) site; dsPAS, extended poly(A) site; avg, average.

To examine the effect of CPSF1-dependent changes in poly(A) site usage on gene expression, we integrated the RNA-seq and PAC-seq datasets. Because the effects of CPSF1 knockdown on PAC-seq read density were most prevalent in the genomic regions spanning 10 kb downstream of annotated 3′ UTRs, we focused our initial analysis on those regions. For each gene, we assessed the relationship between gene expression changes measured by RNA-seq and a PAC-score (poly(A) site cluster score) that reflected the change in PAC-seq read density within the 10 kb downstream sequence region relative to the 3′ UTR sequence region following CPSF1 knockdown (Figure 5A). A higher PAC-score equates to higher usage of poly(A) sites in the 10 kb downstream region vs. the annotated 3′ UTR following CPSF1 knockdown, and a lower PAC-score reflects lower poly(A) site usage of poly(A) sites in the 10 kb downstream region vs. the 3′ UTR following CSPF1 knockdown (Figure 5B).

We observed that the majority of the genes displayed lower expression levels and higher usage of poly(A) sites in the 10 kb downstream region relative to the 3′ UTR following CPSF1 knockdown (quadrant 1 in Figures 5C–5E; Table S4). Assessing PAC-seq read coverage of representative genes in this group (*UGT2B17* in LNCaP cells, *GPI* in LNCaP95 cells, and *ALDH3A2* in 22Rv1 cells) confirmed increased usage of poly(A) sites within the 10 kb downstream region upon CPSF1 knockdown (Figures 5F–5H). The second most abundant group of genes was made up of those displaying higher expression and lower usage of poly(A) sites in the 10 kb downstream sequence region relative to the 3′ UTR sequence region upon CPSF1 knockdown (quadrant 3 in Figures 5C–5E). These results indicate that CPSF1 functions in prostate cancer cells to repress and activate the usage of thousands of poly(A) sites located downstream of annotated 3′ UTRs, which leads to increased gene expression if these sites are repressed and to decreased gene expression if these sites are activated.

We next sought to examine the clinical relevance of these mechanisms identified in prostate cancer cell lines subjected to CPSF1 knockdown. For this, we generated a quadrant I (QI) gene set reflecting the genes that displayed decreased expression and increased usage of poly(A) sites 10 kb downstream of annotated 3′ UTRs after CPSF1 knockdown in at least 2 of the 3 cell lines we analyzed (Table S2). The aggregate expression of genes in this QI gene set correlated positively with CPSF1 mRNA expression in 497 prostate cancer specimens from TCGA-PRAD dataset^42^ (Figure 5I) and in 210 CRPC specimens from the SU2C dataset^43^ (Figure 5J). In TCGA-PRAD dataset, the aggregate expression of this QI gene set was higher in 497 prostate cancer specimens compared to 52 benign prostate specimens (Figure 5K). Further, in the SU2C dataset, the levels of *CPSF1* and the QI gene set displayed a positive correlation with genes in the HALLMARK_GLYCOLYSIS gene set (Figures 5L and 5M). These data indicate that the changes in CPSF1 levels that occur in clinical prostate cancers during disease progression are likely to manifest as gene expression changes in the same genes that we identified in prostate cancer cell lines subjected to CPSF1 knockdown, including genes in the HALLMARK_GLYCOLYSIS gene set. Accordingly, these gene expression changes in clinical prostate cancer specimens are likely to occur via CPSF1-dependent alterations in the use of poly(A) sites located downstream of 3′ UTRs.

To expand on our findings, we also examined the gene expression effects of CPSF1-dependent changes in poly(A) site usage within introns or exons. Similar to our analysis of the 10 kb downstream region (Figures 5A and 5B), for each gene, we assessed the relationship between the PAC-score in the intronic or exonic regions with RNA-seq expression changes. Within intronic regions, we observed that the majority of genes displayed reduced usage of poly(A) sites and increased expression upon CPSF1 knockdown (Figures S5A–S5C; Table S5). Within exonic regions, we observed a small enrichment of genes displaying reduced usage of poly(A) sites and increased expression upon CPSF1 knockdown (Figures S5D–S5F; Table S6). These data suggest that CPSF1 is required for the use of poly(A) sites in introns and exons, which is associated with lower levels of gene expression.

### CPSF1 regulates expression of glycolytic factors by directing poly(A) site usage

**Figure 6.**
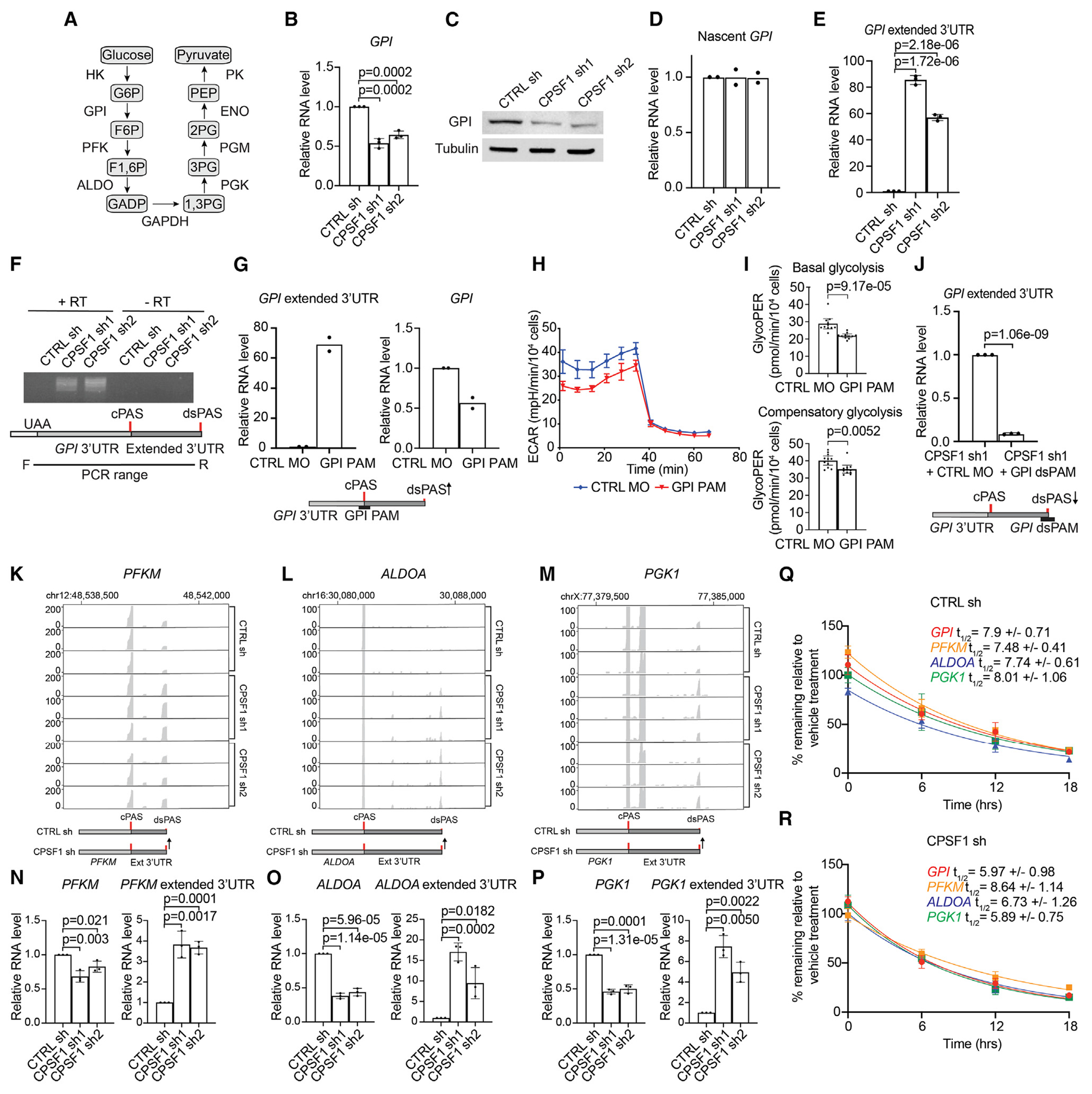
CPSF1 regulates glycolysis by repressing poly(A) site usage distal to annotated 3′ UTRs (A) Illustration of the stepwise conversion of glycolysis to pyruvate. (B–D) Knockdown of CPSF1 decreases GPI mRNA (B) and protein levels (C) but shows no effect on nascent *GPI* mRNA (D). (E and F) qPCR (E) and PCR (F) of the extended 3′ UTR of *GPI* mRNA in CPSF1 shRNA-infected cells. (G) Disrupting the GPI canonical poly(A) site causes a 3′ UTR extension of *GPI* and decreased expression. (H and I) Disrupting the *GPI* canonical poly(A) site lowers ECAR output (H) and basal and compensatory glycolysis (I). (J) Disrupting the downstream poly(A) site of *GPI* in CPSF1-knockdown cells inhibits the extension of the *GPI* 3′ UTR. (K–M) Coverage of PAC-seq reads for *PFKM* (K), *ALDOA* (L), and *PGK1* (M). Red lines denote poly(A) sites called by DPAC analysis. (N–P) qPCR validation of the 3′ UTR extension and decreased expression for *PFKM* (N), *ALDOA* (O), and *PGK1* (P). (Q and R) CTRL shRNA and CPSF1 shRNA cells were treated with actinomycin D (Act-D) and the percentage of remaining mRNA relative to vehicle was quantified by qPCR. For half-life calculations, 6 h post-Act-D treatment was defined as time “0” because no decay was observed from 6 h of treatment. All experiments were performed in LNCaP95 cells. Data are mean with SD. Significance was assessed by unpaired two-sided t tests. RT, reverse transcriptase; cPAS, canonical poly(A) site; dsPAS, downstream poly(A) site; PAM, poly(A) morpholino; t_1/2_, half-life (hours).

To investigate the mechanism by which CPSF1 can repress the usage of poly(A) sites located downstream of 3′ UTRs to promote the expression of those genes, we focused on *GPI* (Figure 5D). *GPI* encodes glucose-6-phosphate isomerase, which catalyzes the second step of glycolysis (Figure 6A). We confirmed that GPI mRNA and protein levels were decreased in LNCaP95 cells upon CPSF1 shRNA knockdown (Figures 6B and 6C). To determine whether this was due to a post-transcriptional mechanism, we performed nascent RNA labeling. The levels of newly transcribed *GPI* mRNAs were unaffected by CPSF1 knockdown in LNCaP95 cells, indicating that transcriptional changes were not the cause of decreased *GPI* mRNA levels (Figure 6D). Next, we tested whether increased usage of a *GPI* downstream poly(A) site was associated with the lengthening of *GPI* transcripts. RT-qPCR with primers targeted upstream of this poly(A) site revealed upregulation, consistent with *GPI* transcript lengthening following CPSF1 knockdown in LNCaP95 cells (Figure 6E). Indeed, RT-PCR with a forward primer located upstream of the stop codon in the terminal exon of *GPI* and a reverse primer near the downstream poly(A) site amplified a product in cells subjected to CPSF1 knockdown but not in control cells (Figure 6F).

To confirm that usage of this poly(A) site located downstream of the annotated *GPI* 3′ UTR was responsible for *GPI* 3′ UTR lengthening, we designed an antisense morpholino to impose a steric blockade of the annotated poly(A) site in the *GPI* 3′ UTR, which we termed GPI poly(A) mask (PAM). LNCaP95 cells transfected with GPI PAM displayed increased levels of longer *GPI* transcripts, decreased overall expression of *GPI*, and decreased basal and compensatory glycolysis (Figures 6G–6I), which matched the effects of CPSF1 knockdown. Conversely, an antisense morpholino designed to mask the poly(A) site located downstream of the annotated *GPI* 3′ UTR (termed dsGPI PAM) reduced the expression of longer *GPI* transcripts in LNCaP95 cells following CPSF1 knockdown (Figure 6J). These data demonstrate that this single poly(A) site located downstream of the annotated *GPI* 3′ UTR regulates the observed 3′ UTR extension event.

*PFKM, ALDOA*, and *PGK1* genes encode phosphofructokinase, aldolase, and phosphoglycerate kinase proteins that regulate central glycolysis (Figure 6A). These genes displayed the same pattern as *GPI*, wherein CPSF1 knockdown decreased mRNA expression and increased PAC-seq read density downstream of the annotated 3′ UTRs (Figures 6K–6M). Using RT-qPCR, we confirmed lower expression of these genes following CPSF1 knockdown in LNCaP95 cells, as well as 3′ UTR lengthening of these transcripts (Figures 6N–6P). Because the inhibition of mRNA cleavage and polyadenylation can lead to transcriptional readthrough,^44–46^ we performed nascent transcript labeling in LNCaP95 cells stably infected with CPSF1 shRNA and found that newly transcribed *GPI, ALDOA, PGK1*, and *PFKM* transcripts displayed 3′ UTR lengthening relative to LNCaP95 cells stably infected with control shRNA (Figure S6A). These results suggest that CPSF1 knockdown promotes transcriptional readthrough of these transcripts. We examined the stability of *GPI, PFKM, ALDOA*, and *PGK1* transcripts in CPSF1-knockdown LNCaP95 cells and found a shortened half-life of each mRNA except *PFKM* (Figures 6Q and 6R). The lack of change in *PFKM* mRNA stability may reflect the smaller expression decrease observed for *PFKM* upon CPSF1 knockdown compared to the other factors (Figures 6N–6P).

To test if coordinate effects of CPSF1 knockdown on multiple genes in the glycolysis pathway may explain the selective inhibition of prostate cancer cell growth, we treated LNCaP95 and RWPE-1 cells with the aldolase inhibitor aldometanib^47^ and the PGK1 inhibitor CBR-470-1.^48^ LNCaP95 and RWPE-1 cell lines displayed similar sensitivity to growth inhibition by these inhibitors when they were tested as single agents (Figures S6B–S6E). However, when these two drugs were tested in combination, there were synergistic effects observed in LNCaP95 cells but not in the benign prostate epithelial cell line RWPE-1 (Figures S6F and S6G). We expanded this analysis to LNCaP and 22Rv1 prostate cancer cells and observed synergistic effects of aldometanib and CBR-470-1 on growth inhibition in these cell lines as well (Figures S6H and S6I). These results indicate that changes in the expression of combinations of genes, as opposed to individual genes, may explain the selective inhibitory effects of CPSF1 shRNA knockdown on the growth of prostate cancer cells but not prostate epithelial cells (Figures 2J–2M).

Overall, these data demonstrate that an outcome of the 3′ UTR lengthening in cells subjected to CPSF1 knockdown is increased decay of mRNA transcripts leading to lower overall expression. These findings identify that a conserved mechanism regulated by CPSF1 is the repression of poly(A) sites downstream of 3′ UTRs to sustain high levels of mRNAs, including mRNAs encoding glycolytic enzymes. CPSF1 as well as mRNAs that are regulated by this mechanism are increased during prostate cancer progression, and the knockdown of CPSF1 inhibits the growth of prostate cancer cells but not benign epithelial prostate cells. Therefore, CPSF1 and CPSF1-regulated mRNAs represent vulnerabilities that could be exploited therapeutically to inhibit glycolysis and the growth of prostate cancer cells.

## DISCUSSION

There has been significant progress in developing therapeutics that improve the overall survival of patients with CRPC. However, despite these advances, CRPC remains a uniformly lethal disease. This demonstrates the need for the identification of new therapeutic targets, which could lead to the development of new treatments for patients with CRPC. In this work, we have identified CPSF1 as a key regulator of global APA in prostate cancer and shown that CPSF1 is required for the growth of androgen-dependent and androgen-independent prostate cancer cell lines. We found that CPSF1 sustains levels of mRNAs encoding glycolysis regulators, highlighting a vulnerability in advanced prostate cancer that is independent of AR and AR-regulated pathways. Our findings that CPSF1 is upregulated in patients with CRPC, that patients with high CPSF1 expression have worse progression-free survival, and that benign prostate epithelial cells display no growth inhibition from CPSF1 knockdown collectively provide strong evidence for the inhibition of CPSF1 as a therapeutic strategy to treat prostate cancer.

Currently, there are no small-molecule inhibitors available that target CPSF1. The small-molecule inhibitor of CPSF3, JTE-607, has been shown to be effective in killing acute myeloid leukemia and Ewing’s sarcoma cells and inhibit breast cancer cell migration and invasion.^16,49^ Our work extends these findings by demonstrating that JTE-607 inhibits the growth of hormone-sensitive and -insensitive prostate cancer cells, as well as benign prostate epithelial cells. JTE-607 is a pro-drug with a carboxylic acid derivative that binds directly to CPSF3. The mode of the JTE-607 carboxylic acid derivative binding to CPSF3 mimics that of pre-mRNA binding to CPSF3, which provides a mechanism for the observed JTE-607-mediated inhibition. Our work showing that CPSF1-regulated genes are targets of JTE-607, but not all targets of JTE-607 are regulated by CPSF1, indicates that applying strategies to develop or identify CPSF1-specific inhibitors could yield attractive, and perhaps more specific, therapeutic candidates for prostate cancer.

An additional strategy for inhibiting CPSF1 in prostate cancer could be to interfere with CPSF1-mediated regulation of target mRNAs. Our work has highlighted *GPI, PFKM, ALDOA*, and *PGK1* as key glycolysis-associated mRNAs that display CPSF1-dependent expression in prostate cancer cell lines. Further, we have shown that an antisense morpholino targeted to the poly(A) site in the *GPI* 3′ UTR produced many of the same effects as CPSF1 knockdown, including *GPI* transcript lengthening via polyadenylation downstream of the 3′ UTR, reductions in GPI levels, and decreased glycolytic output. This antisense targeting strategy applied to a collection of key CPSF1-regulated target mRNAs could be employed to mimic direct CPSF1-inhibition. Potential for this approach is supported by various oligonucleotide-based drugs that are approved for use in the clinic,^50,51^ albeit not for oncology applications. We also found that the small-molecule inhibitors aldometanib and CBR-470-1 display synergy for inhibiting the growth of prostate cancer cells but not benign prostate epithelial cells. These small-molecule inhibitors have not previously been used in prostate cancer cells and could provide another strategy to interfere with CPSF1-mediated regulation.

3′ UTR-shortening events through APA within the canonical 3′ UTR have been well documented in various cancers.^2,4,23^ In this study, we have uncovered a function of poly(A) site suppression by CPSF1, specifically in regions distal to annotated 3′ UTRs. Our data highlight specific factors of the glycolysis pathway as targets of this mechanism of CPSF1 regulation. However, our data also highlight thousands of other genes that are regulated by this mechanism to maintain high expression of shorter 3′ UTR transcript isoforms in prostate cancer cells. CPSF1 has been shown to be required for the growth of multiple cancer subtypes.^12–14^ Our data indicate that CPSF1-mediated poly(A) site repression may be a conserved mechanism that is exploited by various cancers to alter gene expression.

While our work focused primarily on studying intergenic poly(A) sites suppressed by CPSF1, we also identified thousands of intronic CPSF1-regulated poly(A) sites, the majority of which were repressed by CPSF1 knockdown and associated with increased mRNA expression. In previous work, we found that CPSF1 is required for intronic polyadenylation in the *AR* gene, which is a pre-requisite for the splicing of alternative *AR* cryptic exons and the synthesis of truncated, constitutively active AR variants, including AR-V7.^9^ The current study extends this finding by demonstrating that CPSF1-dependent intronic poly(A) site selection is more widespread in prostate cancer cells than recognized from focused studies on *AR*. Overall, our findings that the major effects of CPSF1 in prostate cancer cells are to repress intergenic poly(A) site usage and promote intronic poly(A) site usage reveal intricate mechanisms of gene expression regulation that are required for prostate cancer cell growth.

Although our work defines genome-wide global poly(A) site regulation by CPSF1, the mechanism by which CPSF1 inhibits or supports usage of specific poly(A) sites remains unknown. CPSF1 contains no RNA-binding motif and does not make protein:RNA contacts with the AAUAAA motif in cryo-electron microscopy (cryo-EM) and X-ray crystal structures.^52,53^ Rather, CPSF1 binds to the AAUAAA motif via protein:protein interactions with WDR33 and CPSF4,^52,53^ suggesting that CPSF1 directs poly(A) site usage through non-direct RNA interactions. We demonstrate that transcriptional readthrough of *GPI, PFKM, ALDOA*, and *PGK1* transcripts occurs upon CPSF1 knockdown, which may also be the case for the thousands of other transcripts that are downregulated by CPSF1 knockdown. Future work should aim to define the mechanisms by which CPSF1 regulates usage of the poly(A) sites identified in this study. Understanding the mechanism of CPSF1-regulated poly(A) site selection could reveal sequence elements and factors that maintain a conserved function in poly(A) site cleavage and uncover therapeutic targets that are required for the growth and progression of various cancers.

### Limitations of the study

Our work has several limitations. We performed experiments in prostate cancer cell lines grown *in vitro*, which may not reflect the effects of gene regulation by CPSF1 *in vivo*. We used two independent shRNAs for CPSF1 knockdown, but the efficiency of CPSF1 shRNA knockdown by these shRNAs in different cell lines may not be identical, which could explain some of the differential responses we observed. Although we discovered thousands of poly(A) sites regulated by CPSF1, we have not confirmed if the regulation by CPSF1 is direct or indirect. We have found that CPSF1 knockdown leads to the selective inhibition of prostate cancer cell growth, but we have not identified the specific downstream target(s) of CPSF1 that explain these differential effects. Our PAC-seq dataset revealed thousands of poly(A) sites that are regulated by CPSF1 knockdown, but the vast majority of these sites have not been validated beyond bioinformatic analysis.

## RESOURCE AVAILABILITY

### Lead contact

Requests for further information and resources and reagents should be directed to and will be fulfilled by the lead contact, Scott M. Dehm (dehm@umn.edu).

### Materials availability

All materials are available from the lead contact upon request.

### Data and code availability

- The RNA-seq and PAC-seq data have been deposited at GEO and are publicly available as of the date of publication. The sequencing data are available under the GEO accession numbers GEO: GSE263384 (PAC-seq) and GEO: GSE263385 (RNA-seq).
- This paper does not report original code.
- Any additional information required to reanalyze the data reported in this paper is available from the lead contact upon request.

## STAR★METHODS

### EXPERIMENTAL MODEL AND STUDY PARTICIPANT DETAIL

LNCaP, 22Rv1, and RWPE-1 cells were obtained from American Type Culture Collection (ATCC). LNCaP95 cells were a gift from Jun Luo (Johns Hopkins University). LNCaP and 22Rv1 cells were maintained in Roswell Park Memorial Institute (RPMI) 1640 medium (Gibco) with 10% fetal bovine serum (FBS) and antibiotics (penicillin/streptomycin). RWPE-1 cells were cultured in Keratinocyte-SFM supplemented with 2.5μg EGF, 25mg Bovine Pituitary Extract, and antibiotics (penicillin/streptomycin). LNCaP95 cells were cultured in phenol red–free RPMI 1640 medium supplemented with 10% FBS and penicillin/streptomycin. All cells were grown at 37C and are male sex.

### METHOD DETAILS

#### siRNA and morpholino transfections

For siRNA transfections, 1/6^th^ of the cells growing in a confluent T175 flask were used as input for each electroporation. 22Rv1 and LNCaP95 cells in RPMI 1640 medium supplemented with 10% charcoal-stripped FBS were mixed with 200 pmol targeted or non-targeting control siRNAs (Dharmacon) in a total volume of 400ul. LNCaP cells were transfected in RPMI 1640 medium supplemented with 10% FBS and mixed with 200 pmol targeted or nontargeting control siRNAs (Dharmacon) in a total volume of 400ul. siRNAs were obtained from Dharmacon: nontargeting negative control (D-001210-01), CPSF1 smartpool: M-020395-01-0005, CPSF2 smartpool (M-013404-00-0005), CPSF3 smartpool (M-006365-00-0005), CPSF4 smartpool (M-012292-02-0005), WDR33 smartpool (M-017101-02-0005), FIP1L1 smartpool (M-014670-00-0005), CSTF1 smartpool (M-011245-02-0005), CSTF2 smartpool (M-011246-01-0005), CSTF3 smartpool (M-011247-02-0005), CPSF6 smartpool (M-012334-01-0005), CPSF7 smartpool (M-015842-00-0005), NUDT21 smartpool (M-012335-01-0005), PCF11 smartpool (M-015381-01-0005), CLP1 smartpool (M-019895-00-0005).

For morpholinos, Gene Tools’ design tool was used to design a 25-mer consisting of the sequence 5′ ACCTCCTCCGTGGCATCTTTATTTT 3′ targeted to the GPI canonical poly(A) site (GPI PAM) and an 28-mer consisting of the sequence 5′ AGTTTTTAATTTTTAGAGAAACACACCA 3′ to the extended poly(A) site (extPAM). As a control, a standard commercially available nontargeting control morpholino was used (Gene Tools). For morpholino transfections, 1/6^th^ of LNCaP95 cells growing in a confluent T175 flask were used as input for each electroporation. LNCaP95 cells in RPMI 1640 medium supplemented with 10% FBS were mixed with 10 mmol/L targeting or control morpholino. Cell/siRNA and cells/morpholino mixtures were electroporated in a 4-mm gap-width cuvette (BTX) with a BTX Square Wave Electroporator (LNCaP and LNCaP95: 305 V, 10-ms pulse, 22Rv1: 350 V, 10-ms pulse). Following a 15-min recovery, cells were seeded in appropriate culture medium.

#### Plasmid constructs

The non-silencing shRNA pGIPZ lentiviral vector for shRNA control (CTRL sh) and the pGIPZ shRNA constructs for CPSF1 (V3LHS_638374 and V3LHS_638371) were purchased from Horizon Discovery. pLEX_307 was a gift from David Root (Addgene plasmid # 41392). pENTR1A-GFP-N2 (FR1) was a gift from Eric Campeau & Paul Kaufman (Addgene plasmid # 19364). The CPSF1 cDNA was purchased from Horizon Discovery packaged into vector pENTR221 (OHS5893-202494652). Gateway cloning was used to transfer the CPSF1 cDNA from pENTR221 or GFP from pENTR1A-GFP-N2 into the pLEX_307 vector.

#### Lenitvirus production

For CTRL shRNA, CPSF1 sh1, and CPSF1 sh2 virus production the lentivirus vector, psPAX, and pMD2.G viral packaging vectors were mixed for 5 min in DMEM then mixed with Polyethylenimine (PEI) in DMEM for an additional 5 min. This mixture was then added to 293T cells and virus was harvested day 2, 3, and 4 post-infection and concentrated using Lenti-X concentrator (Takara Bio). For GFP and CPSF1 overexpression lentivirus the CPSF1 or GFP containing pLEX_307 vector was mixed with pPACKH1 and PureFection (System Biosciences), incubated for 15 min, then added to 293T cells and virus was harvested day 2, 3, and 4 post-infection and concentrated using Lenti-X concentrator (Takara Bio).

#### Cell growth assays

Electroporated 22Rv1 cells were seeded at 8,000 cells per well and electroporated LNCaP95 cells were seeded at 8,800 cells per well in 24-well dishes in RPMI 1640 medium supplemented with 10% charcoal-stripped FBS. Electroporated LNCaP cells were seeded at 10,000 cells per well in 24-well dishes in RPMI 1640 medium supplemented with 10% FBS. shRNA infected LNCaP cells were seeded at 8,000 cells per well in RPMI 1640 supplemented with 10% FBS. shRNA infected 22Rv1 and LNCaP95 cells were seeded at 10,000 cells per well in RPMI 1640 supplemented with 10% FBS. shRNA infected RWPE-1 cells were seeded at 4,000 cells per well in 24-well dishes in Keratinocyte-SFM (ThermoFisher) supplemented with 2.5μg EGF and 25mg Bovine Pituitary Extract. LNCaP CPSF1 and GFP overexpression cells were seeded at 8,000 cells in RPMI 1640 supplemented with 10% FBS +/− enzalutamide. At indicated time-points, cells were fixed and stained with crystal violet as previously described,^65^ and the absorbance was measured at 560 nM. For siRNA electroporated cells data are presented as mean ± 95% CI of 6 biological replicates performed as 3 independent experiments. For shRNA infected cells data are presented as mean ± 95% CI of 3 biological replicates performed as 3 independent experiments. Statistical tests to assess the significance of differences between two means were performed using unpaired 2-sided Student’s t-tests. For growth assays performed in 2 mM glucose, glucose-free RPMI 1640 medium (ThermoFisher, 11879020) was supplemented with dialyzed FBS (ThermoFisher, A3382001) and glucose (ThermoFisher, A2494001) was supplemented to a concentration of 2 mM. For metabolite rescue experiments, pyruvate (Agilent, 103578-100), alanine (Sigma, A7627), uridine (Sigma, U3003), nicotinic acid (Sigma, N0761), and oxaloacetic acid (Sigma, O7753) were resuspended in sterile H_2_O and diluted into RPMI 1640 medium supplemented with 10% FBS media at indicated concentrations. Palmitic acid (ThermoFisher, 129702500) was resuspended in absolute ethanol, added to a mix of 11.7 mM pluronic F-68 (Gibco 24040-032), 0.022% Tween-80, and 0.148 mM DL-alpha-Tocopherol prior to diluting in RPMI 1640 medium supplemented with 10% FBS at indicated concentrations.

#### RT-PCR

Total RNA was extracted from cells using a ReliaPrep RNA miniprep kit (Promega) according to manufacturer instructions. Total RNA (1 μg) was reverse transcribed using the qScript cDNA Supermix (Quantabio) according to manufacturer instructions. Quantitative PCR was performed on a Bio-Rad CFX Connect Real-Time Detection System using SYBR Green Master Mix (Bio-Rad) with primers listed in Table S2. 1 μL of cDNA as input in a 20 μL reaction. Cycle thresholds of amplification (Ct) were determined using Bio-Rad CFX manager software. Relative quantification was used to determine fold change in expression levels by the comparative Ct method using the formula 2^−ΔΔCt^ with *ACTB* as calibrator. PCR reactions were performed in technical duplicate with at least two biological replicates.

#### Western blot

Cells were lysed in 1X Laemmli buffer (65mM Tris-HCl, pH7.0, 2%(w/v) SDS, 5% (v/v) β-mercaptoethanol, 10% (v/v) glycerol, and 0.5% (w/v) bromophenol blue) boiled at 100°C for 10 min and homogenized using a 29½-gauge insulin syringe (Comfort Point). Equal protein masses of lysates were separated by electrophoresis in 6.5% polyacrylamide gels for CPSF1 and PCF11 or 10% polyacrylamide gels for CPSF6, NUDT21, and tubulin, followed by transfer to polyvinylidene difluoride membranes (Immobilon-P, Millipore). Membranes were incubated with primary antibodies incubated in milk-TBST overnight at 4°C and the HRP-conjugated secondary antibodies in milk-TBST at room temperature for 1 h. Blots were incubated with Super Signal West Pico (Thermo) or ProSignal Femto (Prometheus) and luminescence was imaged using an iBright CL750 system (Thermo Fisher).

#### Antibodies

Antibodies were purchased from Santa Cruz for western blot detection of CPSF1, (G-10, sc-166281, 1:100) and tubulin (B-5-1-2, sc-23948, 1:3000), Proteintech for western blot detection of NUDT21 (66335-1-Ig, 1:500) and PCF11 (23540-1-AP, 1:500), Fortis Life Sciences for western blot detection of CPSF6 (A301-356A, 1:1,000) and Origene for western blot detection of GPI (TA501171, 1:500).

#### Progression-free survival analysis

Curated progression-free interval (PFI) data for prostatic adenocarcinoma (PRAD) specimens were obtained from a published The Cancer Genome Atlas (TCGA) resource of pan-cancer clinical data.^33^ For each indicated gene, patients in the top vs. bottom quartiles of gene expression extracted from TCGA RNA-seq data were tested for differences in PFI using Kaplan-Meier survival analysis with the R packages survival^54,66^ and survminer.^55^ *p*-values were considered significant if *p* < 0.05.

#### Analysis of differential gene expression in mCRPC vs. primary prostate cancer

Differential expression of genes between mCRPC vs. primary prostate cancer samples were analyzed across three publicly available microarray datasets^30–32^ GEO: GSE35988, GEO: GSE21034, and GEO: GSE3325. Differential gene expression analysis was performed independently to compare mCRPC vs. primary prostate cancer samples for each GEO dataset using the GEOquery^56^ and limma^57^ R packages. Log2 fold change (log2FC) expression values are reported and annotated based on a significance threshold of adjusted *p*-value <0.05.

#### RNA-seq

For RNA-seq, 1 μg of total RNA extracted from three biological replicates of CTRL shRNA, CPSF1 shRNA 1, and CPSF1 shRNA 2 infected cells were submitted to University of Minnesota Genomics Center. The library was prepared with TruSeq Stranded mRNA kit (Illumina) according to manufacturer’s instructions and was constructed for 2 × 150 paired-end sequencing on an Illumina NovaSeq 6000 system.

#### Identification of differentially expressed genes in RNA-seq data

Fastq files containing 75 bp paired-end reads were aligned to the hg19 reference genome using HiSat2 (v. 2.1.0).^58^ Subread (v. 2.8.2)^59^ was used to quantify gene expression using the version 87 GRCh37 annotation from Ensembl.^60^ Count data were generated using Subread’s featureCounts() command with the “strandSpecific” parameter set to 2 for opposite-stranded libraries. Genes were filtered to include only genes that had a cpm (counts per million) value greater than 2 cpm across all experimental conditions per cell line. Differential expression testing was performed in edgeR (v. 3.36.0)^61,67^ using a generalized linear model and a quasi-likelihood *F*-test to compare the three control samples to the six siRNA samples for each cell line. The Benjamini–Hochberg method was used for multiple hypothesis testing correction. An adjusted *p*-value = 0.05 was used as a differential expression significance threshold. Differential gene expression tables are in Table S1.

#### Gene set enrichment analysis

The shCPSF1_DOWN and shCPSF1_UP gene sets were generated from the intersecting genes that were the most highly downregulated (−log10 FDR < −4) or up-regulated (−log10 FDR >4) in RNA-seq data from LNCaP, LNCaP95, and 22Rv1 cells stably expressing CPSF1 shRNA vs. control shRNA. Similarly, the JTE-607_UP and JTE-607_DOWN gene sets were generated from the intersecting genes that were the most highly up-regulated (−log10 FDR >4) or downregulated (−log10 FDR < −4) in RNA-seq data from HepG2 and HeLa cells treated with 1 μM and 10 μM of JTE-607 vs. vehicle control (GEO: GSE218557). shCPSF1_DOWN, shCPSF1_UP, JTE-607_DOWN, and JTE-607_UP gene sets are in Table S2. Gene Set Enrichment Analysis (GSEA) was performed using the java implementation of GSEA (v. 4.3.2).^62^ Gene expression datasets were from LNCaP95 and 22Rv1 cells stably expressing CPSF1 shRNA vs. control shRNA, or from HepG2 and HeLa cells treated with 1 μM and 10 μM of JTE-607 vs. vehicle control.^35^ Gene expression datasets were preranked based on the magnitude and direction of unadjusted *p*-values such that the most statistically significant up- and downregulated genes fell at opposite ends of the list. False discovery rate calculations were performed using 10,000 permutations and a seed set to 149 to enable reproducibility. Ranked gene lists were compared against the hallmark gene sets and oncogenic gene sets (C6 gene sets) from MSigDb (v. 2023.1.Hs),^34^ or against the custom gene sets CPSF1_DOWN, CPSF1_UP, JTE_UP, and JTE_DOWN.

#### Glycolytic rate assay

For the Glycolytic Rate Assay, 1.5 × 10^4^ cells/well were seeded in a 96-well XF cell culture microplate in culture medium 24 h before assay (48 h for LNCaP95 cells). ECAR and OCR were measured with an XF96 analyzer in XF base medium (pH 7.4) containing 10mM glucose, 1 mM pyruvate, 2 mM glutamine before and following sequential additions of oligomycin (0.5 μM) and 2-DG (50 mM). Data were analyzed using the Seahorse XF Glycolytic Rate Assay Report Generator package with results normalized against cell number determined by an Agilent BioTek Cytation 1 Cell Imaging Multimode Reader. GlycoPER was calculated using Wave desktop software, using data from the Seahorse glycolytic rate assay.

#### IC50 and drug synergy analysis

LNCaP cells were seeded at 10,000 cells per well in RPMI 1640 medium supplemented with 10% FBS. 22Rv1 cells were seeded at 7,500 cells were well in RPMI 1640 medium supplemented with 10% FBS. LNCaP95 cells were seeded at 15,000 cells per well in RPMI 1640 medium supplemented with 10% FBS. RWPE-1 cells were seeded at 3,000 cells per well in 24-well dishes in Keratinocyte-SFM medium (ThermoFisher) supplemented with 2.5 μg EGF and 25 mg Bovine Pituitary Extract. JTE-607 (MedChemExpress, HY-110133) dissolved in DMSO was added at indicated concentrations, along with DMSO as a vehicle control. After 7 days of drug/vehicle exposure, cells were fixed, stained with crystal violet, and the absorbance was measured at 560 nM IC50 calculations were performed in GraphPad Prism using a nonlinear regression curve. IC50 values were calculated for 9 individual biological replicates and the average +/− the 95% CI were shown. For drug synergy assays, indicated concentrations of aldometanib (MedChemExpress, HY-148189), CBR-470-1 (MedChemExpress, HY-134205A), or vehicle control (DMSO) were added when cells were seeded. After 7 days of drug/vehicle exposure, cells were fixed, stained with crystal violet, and the absorbance was measured at 560 nM. Synergy was analyzed using the SynergyFinder+ program^68^ using the Bliss independence model.^69^

#### PAC-seq library preparation and analysis

Poly(A)-ClickSeq (PAC-seq) was used to investigate APA usage in a genome-wide fashion.^38,39^ 1 μg of total RNA extracted from three biological replicates of CTRL shRNA, CPSF1 shRNA 1, and CPSF1 shRNA 2 infected cells were submitted to ClickSeq Technologies (Galveston, TX) for downstream processing. Briefly, samples were reverse transcribed with partial p7 adaptor (Illumina_4N_21T) and dNTPs with the addition of spiked-in azido-nucleotides (AzVTPs) at 5:1. The p5 adaptor (IDT) was Click-ligated to 5′ end of cDNA by CuAAC. The libraries were amplified using a universal primer and 3′ indexing primer, followed by purification on a 2% agarose gel with extraction of the 200–300 bp amplicon. Barcoded libraries were pooled and sequenced on an Illumina NextSeq550 with a Mid Output 400M, v2 kit. The reads were processed and quality filtered as previously described^38,39^ and mapped to the human genome (hg19) using Hisat2.^58^ PAC-seq data were analyzed with the differential poly(A) clustering DPAC pipeline v1.2^40^ using the exon-centric approach, with the –P –M –C –A –B and –D options. The results were filtered such that genes or exons required a minimum of at least 5 mapped reads and with at least 25 A’s in the read. To minimize signal from internal A-rich sequences, the DPAC pipeline removes poly(A) site calls from regions of where the corresponding genomic DNA has more than 12 As in the 20 nucleotides downstream of where the poly(A) site was detected. Alternative polyadenylation (APA) was reported when a gene has two or more PACs with at least one PAC undergoing differential usage with an IHW padj <0.1 and resulting in a fractional change of the PAC usage by at least 10%. The DPAC output tables are in Table S3.

#### Differential PAC usage outside annotated 3′UTRs

To compare PAC usage across annotated 3′ UTRs, CDS, introns, and regions within 10 kb downstream of terminal annotated UTRs, we generated.bed files for each feature type. CDS and 3′ UTR features were pulled from transcripts of protein-coding genes in the Ensembl GRCh37 v87 annotation and filtered to exclude transcripts annotated as “retained_intron” transcripts. To account for polyA sites just outside of annotated 3′ UTRs, each 3′ UTR feature was extended by 300 base pairs in the 3′ direction. Intronic annotations were obtained by subtracting CDS and 3′ UTR features from the full gene features on the same DNA strand using the subtract function in bedtools (v2.30.0).^63^ Regions downstream of terminal annotated UTRs were generated by appending 10 kb onto the 3′ end of gene features, then subtracting same strand exons and 3′ UTRs features. Single-end Illumina PAC-seq libraries were processed to remove polyA sequences using the DPAC pipeline v1.2,^40^ then quantified across these feature types as described for the RNA-seq libraries above with two exceptions. To account for the same-stranded PAC-seq library preparation, “strandSpecific” was set to 1 when running featureCounts(), and “read2pos” was set to 3 so as to assign PAC-seq reads to features based on the location of the 3′-most base. Volcano plots and density plots were generated with ggplot2 (v3.4.2)^64^ using geom_point() and geom_hex() respectively with a minimum expression cutoff of two counts per million in each of the datasets used to generate the plot. Differential PAC usage tables are in Table S3.

#### Integrative analysis of RNA-seq and PAC-seq datasets

PAC-score was determined by subtracting the log2FC PAC counts of intronic, CDS, or 10 kb downstream of terminal annotated 3′ UTR regions from the log2FC PAC counts of the annotated 3′ UTR. Density plots represent log2FC values from differential expression analysis of the RNA-seq dataset and PAC-score values per region from the PAC-seq dataset. Genes represented in the density plots must be quantifiable by all metrics plotted (i.e., log2FC intronic from PAC-seq, log2FC annotated 3′ UTR from PAC-seq, log2FC exons from RNA-seq). Quadrant count tables are in Tables S4–S6.

#### Analysis of CPSF1 and CPSF1 target gene expression in public RNA-seq datasets from clinical prostate cancers

We developed the shCPSF1_Lengthened_DOWN (Quadrant I) gene set based on the following characteristics in RNA-seq and PAC-seq datasets in at least two of the three cell lines (Tables S1 and S4): 3′ UTR lengthening in CPSF1 sh (PAC-score greater than 0.5 log2FC) and decreased RNA-seq expression in CPSF1 sh (CPSF1 sh:Control < −0.25 log2FC). We validated the shCPSF1_Lengthened_DOWN gene set by comparing the expression of these genes against *CPSF1* in publicly available gene expression datasets. For this, the average of the Z-scores of all genes in the shCPSF1_Lengthened_DOWN gene set was compared to the *CPSF1 Z* score using publicly available TCGA-PRAD cohorts (containing 497 specimens, 497 primary, 52 matched normal) and SU2C cohorts^43^ (containing 210 patients). The expression of these genes in benign prostate tissue (*n* = 52) relative to prostate cancer (*n* = 497) was evaluated by comparing average Z-scores of this gene set in public TCGA RNA-seq data from prostate cancer patients. For comparison of the HALLMARK_GLYCOLYSIS gene set to *CPSF1* and the shCPSF1_Lengthened_DOWN gene set, the average *Z* score of genes in the HALLMARK_GLYCOLYSIS gene set from MSigDb (v. 2023.1.Hs)^34^ was compared against the average *Z* score of *CPSF1* or genes in the shCPSF1_Lengthened_DOWN gene set in SU2C cohorts.^43^ R values represent the Pearson correlation and *p*-values were calculated by testing Spearman correlations of the data. The shCPSF1_Lengthened_DOWN gene set is in Table S2.

#### Nascent mRNA capture

Nascent transcripts were labeled with biotin and subjected to streptavidin pull-down using the Click-iT Nascent RNA Capture Kit (Life Technologies) according to manufacturer specifications. Briefly, LNCaP95 cells stably infected with CPSF1 shRNA or CTRL shRNA were pulsed with 5-ethynyl Uridine (5EU) for 1 h to label nascent transcripts, then harvested using ReliaPrep RNA Cell Miniprep columns (Promega). Total RNA was then subjected to a Click chemistry reaction which attached a biotin molecule to 5EU-labeled nascent transcripts. RNA was precipitated, then bound to streptavidin-conjugated magnetic beads and washed 10× to remove unlabeled transcripts, leaving only biotin-5EU-labeled nascent RNA attached to the beads. First-strand cDNA synthesis was performed directly on RNA:bead conjugates using the qScript cDNA Supermix (Quantabio) according to manufacturer specifications, then subjected to qRT-PCR.

#### Actinomycin D assay

CPSF1 shRNA or CTRL shRNA infected LNCaP95 cells were incubated with DMSO as vehicle control or 10 μg/mL Actinomycin D (Act-D) (Sigma) for 6, 12, 18, and 24 h. RNA was harvested using ReliaPrep RNA Cell Miniprep columns (Promega) at the indicated time points and analyzed via qRT-PCR. Relative mRNA levels were generated using cDNA dilutions from DMSO-treated cells, and plotting a standard curve to convert Ct values from Actinomycin D treated cells into%remaining values. Because no decay occurred for each factor by 6 h of Act-D treatment, half-life calculations were performed setting the 6 h Act-D treatment as the “0” time. Half-lifes for each factor were calculated with the equation t_1/2_ = ln(2)/K, using a nonlinear regression model and the one phase decay equation on GraphPad (Prism) to define the decay constant (K).

### QUANTIFICATION AND STATISTICAL ANALYSIS

All statistical analyses were conducted using GraphPad Prism 10 (GraphPad software Inc.). Statistical differences between 2 populations were calculated by unpaired t test (2-tailed). *p* < 0.05 was considered statistically significant. Statistical details of experiments and exact value of n can be found in the figure legends.

## Supplementary Material

1

2

3

4

5

6

7

## Figures and Tables

**Table:** KEY RESOURCES TABLE

REAGENT or RESOURCE	SOURCE	IDENTIFIER
Antibodies		
CPSF1	Santa Cruz Biotechnology	RRID:AB_2084362
tubulin	Santa Cruz Biotechnology	RRID:AB_628410
NUDT21	ProteinTech	RRID:AB_2881715
PCF11	ProteinTech	RRID:AB_2879293
CPSF6	Fortis Life Sciences	RRID:AB_937781
GPI	Origene	RRID:AB_11129604
Chemicals, peptides, and recombinant proteins		
JTE-607	MedChemExpress	HY-110133
Aldometanib	MedChemExpress	HY-148189
CBR-470-1	MedChemExpress	HY-134205A
Critical commercial assays		
XF Glycolytic Rate Assay Kit	Agilent	103344–100
Click-iT Nascent RNA Capture Kit	Life Tech	C10365
Deposited data		
PAC-seq CPSF1 shRNA data	This study	GEO: GSE263384
RNA-seq CPSF1 shRNA data	This study	GEO: GSE263385
Experimental models: Cell lines		
LNCaP	ATCC	NA
22Rv1	ATCC	NA
LNCaP95	Jun Luo (JHU)	NA
RWPE-1	ATCC	NA
Oligonucleotides		
CPSF1 siRNA smartpool	Dharmacon	M-020395-01-0005
CPSF2 siRNA smartpool	Dharmacon	M-013404-00-0005
CPSF3 siRNA smartpool	Dharmacon	M-006365-00-0005
CPSF4 siRNA smartpool	Dharmacon	M-012292-02-0005
WDR33 siRNA smartpool	Dharmacon	M-017101-02-0005
FIP1L1 siRNA smartpool	Dharmacon	M-014670-00-0005
CSTF1 siRNA smartpool	Dharmacon	M-011245-02-0005
CSTF2 siRNA smartpool	Dharmacon	M-011246-01-0005
CSTF3 siRNA smartpool	Dharmacon	M-011247-02-0005
CPSF6 siRNA smartpool	Dharmacon	M-012334-01-0005
CPSF7 siRNA smartpool	Dharmacon	M-015842-00-0005
NUDT21 siRNA smartpool	Dharmacon	M-012335-01-0005
PCF11 siRNA smartpool	Dharmacon	M-015381-01-0005
CLP1 siRNA smartpool	Dharmacon	M-019895-00-0005
GPI PAM morpholino	GeneTools	N/A
extPAM morpholino	GeneTools	N/A
Recombinant DNA		
CTRL shRNA	Horizon discovery	RHS4346
CPSF1 shRNA 1	Horizon discovery	V3LHS_638374
CPSF1 shRNA 2	Horizon discovery	V3LHS_638371
EF1a overexpression vector	Addgene	pLEX_307
CPSF1 cDNA	Horizon Discovery	OHS5893-202494652
GFP gateway cloning vector	Addgene	pENTR1A-GFP-N2
pMD2.G virus packaging vector	–	N/A
psPAX2 virus packaging vector	–	N/A
Software and algorithms		
DPAC pipeline v1.2	Routh et al.^40^	–
GraphPad Prism 10	GraphPad Software	https://www.graphpad.com
IGV 2.16.2	IGV	https://igv.org
R	R Project	https://www.r-project.org
R package survival	Therneau et al.^54^	https://cran.r-project.org/web/packages/survival/index.html
R package survminer	Kassambara et al.^55^	https://CRAN.R-project.org/package=survminer
SynergyFinder	Netphar	https://synergyfinder.org/#!/
R package GEOquery	Davis et al.^56^	https://www.bioconductor.org/packages/release/bioc/html/GEOquery.html
R package limma	Ritchie et al.^57^	https://bioconductor.org/packages/release/bioc/html/limma.html
HiSat2 (v. 2.1.0)	Kim et al.^58^	http://daehwankimlab.github.io/hisat2/download/
Subread (v. 2.8.2)	Liao et al.^59^	https://bioconductor.org/packages/release/bioc/html/Rsubread.html
Ensembl	Cunningham et al.^60^	https://www.ensembl.org/index.html
edgeR (v. 3.36.0)	Robinson et al.^61^	https://bioconductor.org/packages/release/bioc/html/edgeR.html
GSEA (v. 4.3.2)	Subramanian et al.^62^	https://www.gsea-msigdb.org/gsea/index.jsp
MSigDb (v. 2023.1.Hs)	Liberzon et al.^34^	https://www.gsea-msigdb.org/gsea/index.jsp
bedtools (v2.30.0)	Quinlan et al.^63^	https://bedtools.readthedocs.io
ggplot2 (v3.4.2)	Wickham H^64^	https://ggplot2.tidyverse.org

## Highlights

- CPSF1 is upregulated in patients with advanced prostate cancer
- CPSF1 is required for the growth and glycolytic output of prostate cancer cells
- CPSF1 knockdown promotes usage of intergenic poly(A) sites distal to 3′ UTRs
- Antisense targeting of a CPSF1-dependent poly(A) site in *GPI* inhibits glycolysis
