# Supplementary material for: CPSF1 inhibition promotes widespread use of intergenic polyadenylation sites and impairs glycolysis in prostate cancer cells
Source: Cell Rep. Author manuscript; Available in PMC 2025 Feb 17. (PMC11831233; doi:10.1016/j.celrep.2024.115211)
Supplement: 1 [file NIHMS2052781-supplement-1.pdf]

Cell Reports, Volume *44*

Supplemental information

CPSF1 inhibition promotes widespread use of intergenic polyadenylation sites and impairs glycolysis in prostate cancer cells

Kiel T. Tietz, Braedan M. McCluskey, Conor R. Miller, Yingming Li, Sarah A. Munro, and Scott M. Dehm

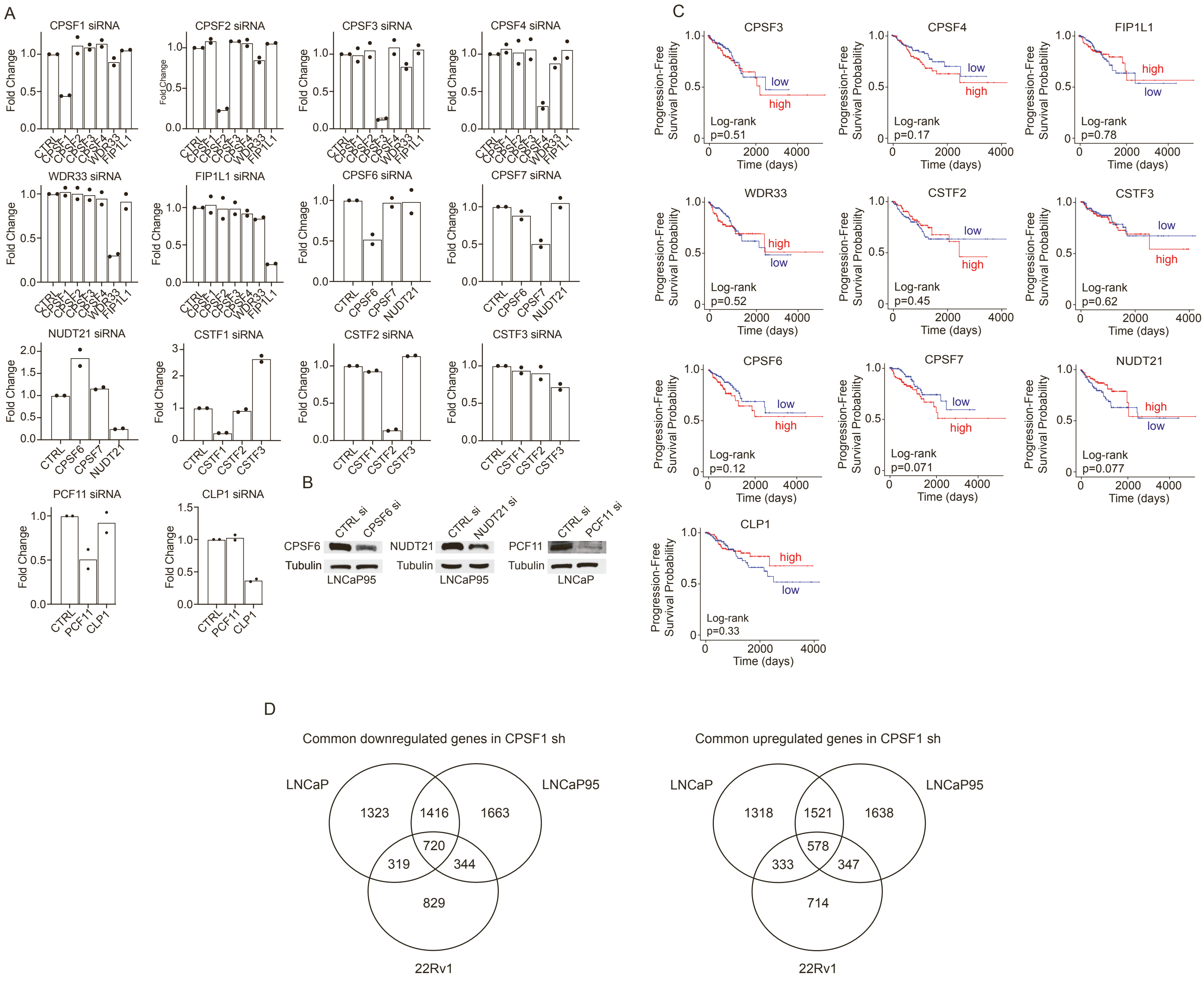
A
CPSF1 siRNA
CPSF2 siRNA
CPSF3 siRNA
CPSF4 siRNA
WDR33 siRNA
FIP1L1 siRNA
CPSF6 siRNA
CPSF7 siRNA
NUDT21 siRNA
CSTF1 siRNA
CSTF2 siRNA
CSTF3 siRNA
PCF11 siRNA
CLP1 siRNA
Fold Change
CTRL
CPSF1
CPSF2
CPSF3
CPSF4
WDR33
FIP1L1
CPSF6
CPSF7
NUDT21
CSTF1
CSTF2
CSTF3
PCF11
CLP1
B
CTRL si
CPSF6 si
NUDT21 si
PCF11 si
CPSF6
NUDT21
PCF11
Tubulin
LNCaP95
LNCaP
C
Progression-Free Survival Probability
Time (days)
CPSF3 Log-rank p=0.51
CPSF4 Log-rank p=0.17
FIP1L1 Log-rank p=0.78
WDR33 Log-rank p=0.52
CSTF2 Log-rank p=0.45
CSTF3 Log-rank p=0.62
CPSF6 Log-rank p=0.12
CPSF7 Log-rank p=0.071
NUDT21 Log-rank p=0.077
CLP1 Log-rank p=0.33
low
high
D
Common downregulated genes in CPSF1 sh
LNCaP
LNCaP95
22Rv1
1323
1416
1663
720
319
344
829
Common upregulated genes in CPSF1 sh
LNCaP
LNCaP95
22Rv1
1318
1521
1638
578
333
347
714

Figure S1: Validation of knockdown in siRNA screen, the association of mRNAs encoding polyadenylation factors with progression-free survival, and common differentially expressed genes in LNCaP, LNCaP95, 22Rv1 cells infected with CPSF1 shRNA. A, RT-qPCR analysis examining the expression of CPSF1, CPSF2, CPSF3, CPSF4, WDR33, FIP1L1, CPSF6, CPSF7, NUDT21, CSTF1, CSTF2, CSTF3, PCF11, and CLP1 mRNAs following transfection of LNCaP95 cells with the indicated siRNA relative to a non-targeting siRNA (CTRL). Data represent mean of 2 biological replicates (n = 2). **B**, Western blot of CPSF6 and NUDT21 in LNCaP95 cells, and PCF11 in LNCaP cells following transfection with indicated siRNAs. **C**, For each indicated gene, patients in the top vs bottom quartiles of gene expression were tested for associations with progression-free survival using TCGA RNA-seq data. Significance was assessed by unpaired 2-sided t-tests. **D**, Venn diagrams illustrating the number of shared genes that are downregulated and upregulated upon CPSF1 shRNA knockdown in LNCaP, LNCaP95 and 22Rv1 cells.

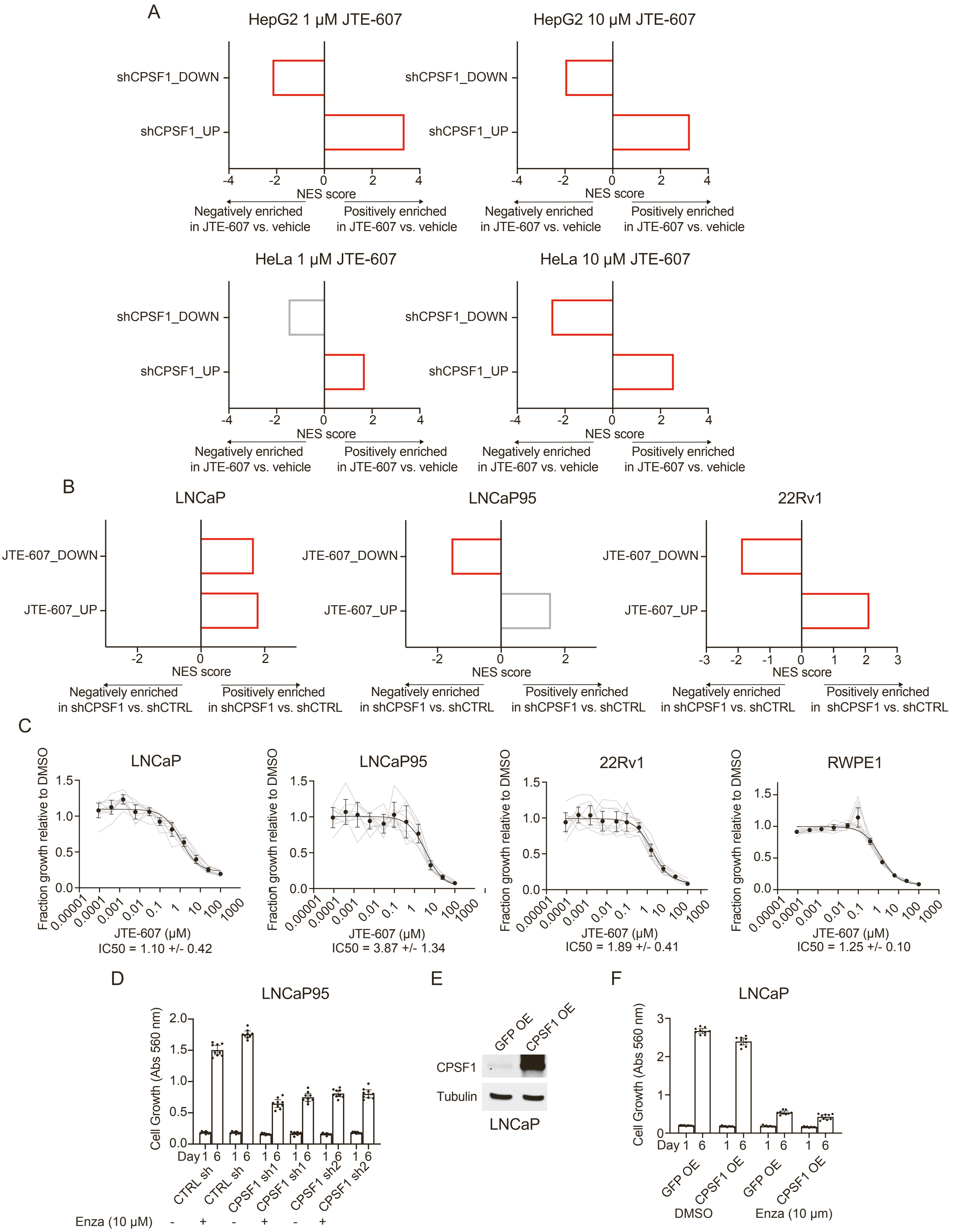

A
HepG2 1 μM JTE-607
HepG2 10 μM JTE-607
HeLa 1 μM JTE-607
HeLa 10 μM JTE-607
shCPSF1_DOWN
shCPSF1_UP
NES score
Negatively enriched in JTE-607 vs. vehicle
Positively enriched in JTE-607 vs. vehicle
B
LNCaP
LNCaP95
22Rv1
JTE-607_DOWN
JTE-607_UP
NES score
Negatively enriched in shCPSF1 vs. shCTRL
Positively enriched in shCPSF1 vs. shCTRL
C
LNCaP
LNCaP95
22Rv1
RWPE1
Fraction growth relative to DMSO
JTE-607 (μM)
IC50 = 1.10 +/- 0.42
IC50 = 3.87 +/- 1.34
IC50 = 1.89 +/- 0.41
IC50 = 1.25 +/- 0.10
D
LNCaP95
Cell Growth (Abs 560 nm)
Day
CTRL sh
CPSF1 sh1
CPSF1 sh2
Enza (10 μM)
E
GFP OE
CPSF1 OE
CPSF1
Tubulin
LNCaP
F
LNCaP
Cell Growth (Abs 560 nm)
Day
GFP OE
CPSF1 OE
DMSO
Enza (10 μm)

Figure S2: CPSF1-regulated genes are distinct from general inhibition of mRNA cleavage and polyadenylation and CPSF1 is not a key determinant of enzalutamide sensitivity in prostate cancer cells. A, Normalized enrichment scores of shCPSF1_DOWN and shCPSF1_UP gene sets in RNA-seq gene expression datasets generated from HepG2 or HeLa cells treated with 1 μM or 10 μM of JTE-607 (vs. DMSO as vehicle control) as indicated. **B**, Normalized enrichment scores of JTE-607_DOWN and JTE-607_UP gene sets in RNA-seq gene expression datasets generated from LNCaP, LNCaP95, or 22Rv1 cells stably infected with lentivirus encoding CPSF1 shRNA (vs. control shRNA). Signatures are red if FDR < 0.05. **C**, LNCaP, LNCaP95, 22Rv1, and RWPE-1 cells were treated with JTE-607 at indicated concentrations and the fraction of growth relative to cells treated with DMSO (as vehicle control) was measured by crystal violet staining on day 7 after treatment was initiated. Gray lines indicate individual biological replicates black lines indicate the average of n = 9 biological replicates +/- 95% CI. The average IC50 of n = 9 is displayed +/- the 95% CI. **D**, Growth of LNCaP95 cells infected with lentivirus encoding shRNAs targeting CPSF1 (CPSF1 sh1 and CPSF1 sh2) or a non-targeting shRNA (CTRL sh) in media containing DMSO or 10 μM enzalutamide. Cell growth was measured on Days 1 and 6 post-seeding using crystal violet staining. **E**, Western blot of CPSF1 (top) and tubulin (bottom) in LNCaP cells infected with GFP or CPSF1 overexpression lentivirus. **F**, Growth of LNCaP cells infected with GFP or CPSF1 overexpression lentivirus in media containing DMSO or 10 μM enzalutamide. Cell growth was measured on Days 1 and 6 post-seeding using crystal violet staining. Data are mean +/- 95% CI. Significance was assessed by unpaired 2-sided t-tests. OE = overexpression. Abs = absorbance. Enza = enzalutamide.

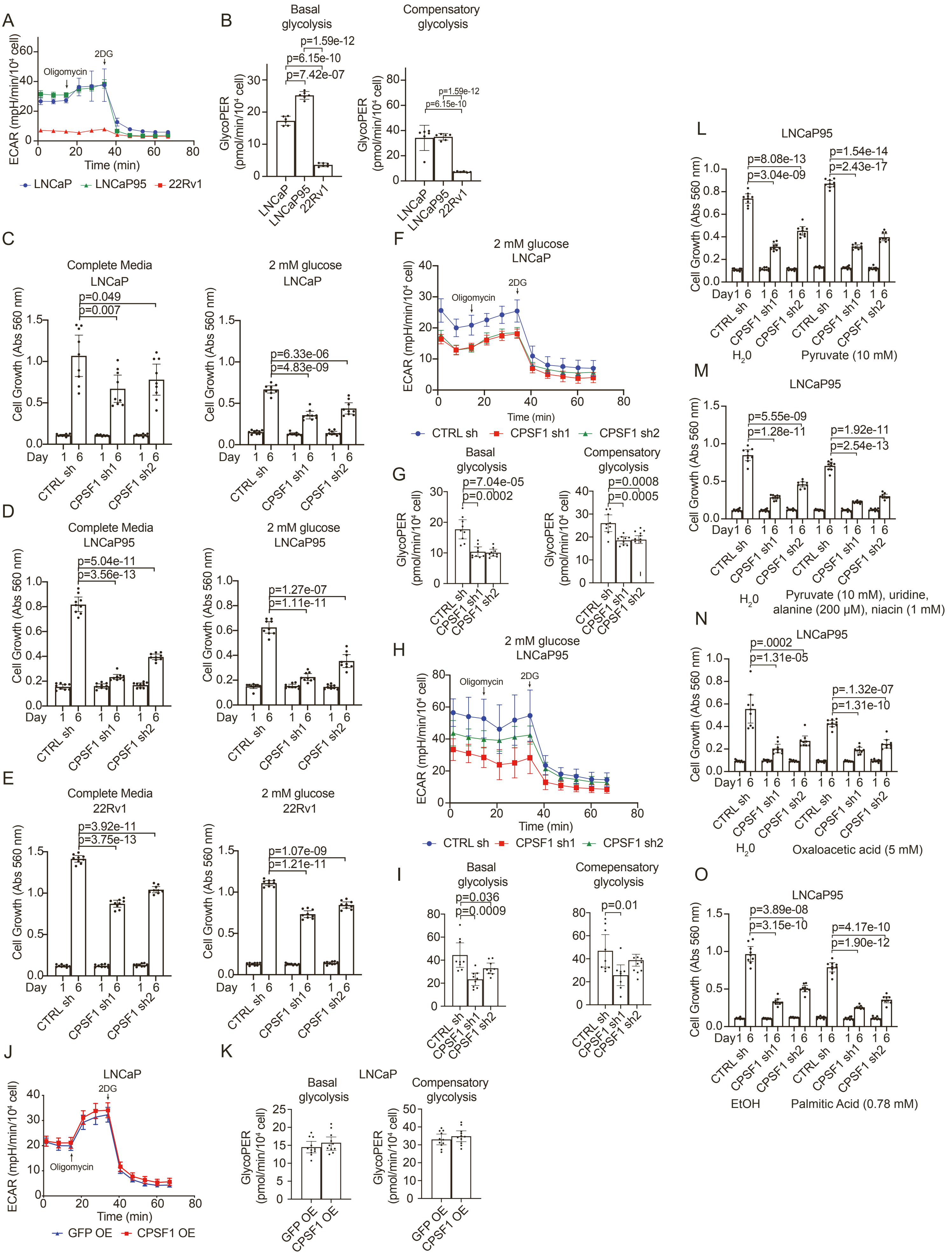
A
Oligomycin
2DG
ECAR (mpH/min/10⁴ cell)
Time (min)
LNCaP
LNCaP95
22Rv1
B
Basal glycolysis
Compensatory glycolysis
GlycoPER (pmol/min/10⁴ cell)
p=1.59e-12
p=6.15e-10
p=7.42e-07
LNCaP
LNCaP95
22Rv1
C
Complete Media LNCaP
2 mM glucose LNCaP
Cell Growth (Abs 560 nm)
p=0.049
p=0.007
p=6.33e-06
p=4.83e-09
Day
CTRL sh
CPSF1 sh1
CPSF1 sh2
D
Complete Media LNCaP95
2 mM glucose LNCaP95
p=5.04e-11
p=3.56e-13
p=1.27e-07
p=1.11e-11
E
Complete Media 22Rv1
2 mM glucose 22Rv1
p=3.92e-11
p=3.75e-13
p=1.07e-09
p=1.21e-11
F
2 mM glucose LNCaP
Oligomycin
2DG
Time (min)
CTRL sh
CPSF1 sh1
CPSF1 sh2
G
Basal glycolysis
Compensatory glycolysis
p=7.04e-05
p=0.0002
p=0.0008
p=0.0005
H
2 mM glucose LNCaP95
I
Basal glycolysis
Comepensatory glycolysis
p=0.036
p=0.0009
p=0.01
J
LNCaP
GFP OE
CPSF1 OE
K
LNCaP
Basal glycolysis
Compensatory glycolysis
GFP OE
CPSF1 OE
L
LNCaP95
p=8.08e-13
p=3.04e-09
p=1.54e-14
p=2.43e-17
H₂0
Pyruvate (10 mM)
M
LNCaP95
p=5.55e-09
p=1.28e-11
p=1.92e-11
p=2.54e-13
H₂0
Pyruvate (10 mM), uridine, alanine (200 μM), niacin (1 mM)
N
LNCaP95
p=.0002
p=1.31e-05
p=.1.32e-07
p=1.31e-10
H₂0
Oxaloacetic acid (5 mM)
O
LNCaP95
p=3.89e-08
p=3.15e-10
p=4.17e-10
p=1.90e-12
EtOH
Palmitic Acid (0.78 mM)

Figure S3: CPFS1 knockdown inhibits prostate cancer cell growth and glycolysis in physiological concentrations of glucose and addition of metabolites do not rescue cell growth inhibition caused by CPSF1 knockdown. A, ECAR output and **B**, Basal and compensatory glycolysis of LNCaP, LNCaP95, and 22Rv1 cells. **C-E**, Growth of LNCaP, LNCaP95, and 22Rv1 cells infected with lentivirus encoding shRNAs targeting CPSF1 (CPSF1 sh1 and CPSF1 sh2) or a non-targeting shRNA (CTRL sh) in either complete media or media containing 2 mM glucose. **F**, ECAR output and **G**, Basal and compensatory glycolysis of CPSF1 shRNA or CTRL shRNA infected LNCaP cells in 2 mM glucose media. **H**, ECAR output and **I**, Basal and compensatory glycolysis of CPSF1 shRNA or CTRL shRNA infected LNCaP95 cells in 2 mM glucose media. **J**, ECAR output and **K**, Basal and compensatory glycolysis of LNCaP cells infected with CPSF1 or GFP overexpression lentivirus. **L**, Growth of LNCaP95 cells infected with CPSF1 shRNA or CTRL shRNA in media containing H_2O or 10 mM pyruvate. **M**, Growth of LNCaP95 cells infected as in panel L in media containing H_2O or 10 mM pyruvate, 200 μM uridine, 200 μM alanine, 1 mM niacin. **N**, Growth of LNCaP95 cells infected as in panel L in media containing H_2O or 5 mM oxaloacetic acid. **O**, Growth of LNCaP95 cells infected as in panel L in media containing ethanol or 0.78 mM palmitic acid. ECAR = extracellular acidification rate, GlycoPER = glycolytic proton efflux rate. All cell growth was measured on Days 1 and 6 post-seeding using crystal violet staining. Data are mean +/- 95% CI. Significance was assessed by unpaired 2-sided t-tests. Abs = absorbance. EtOH = ethanol.

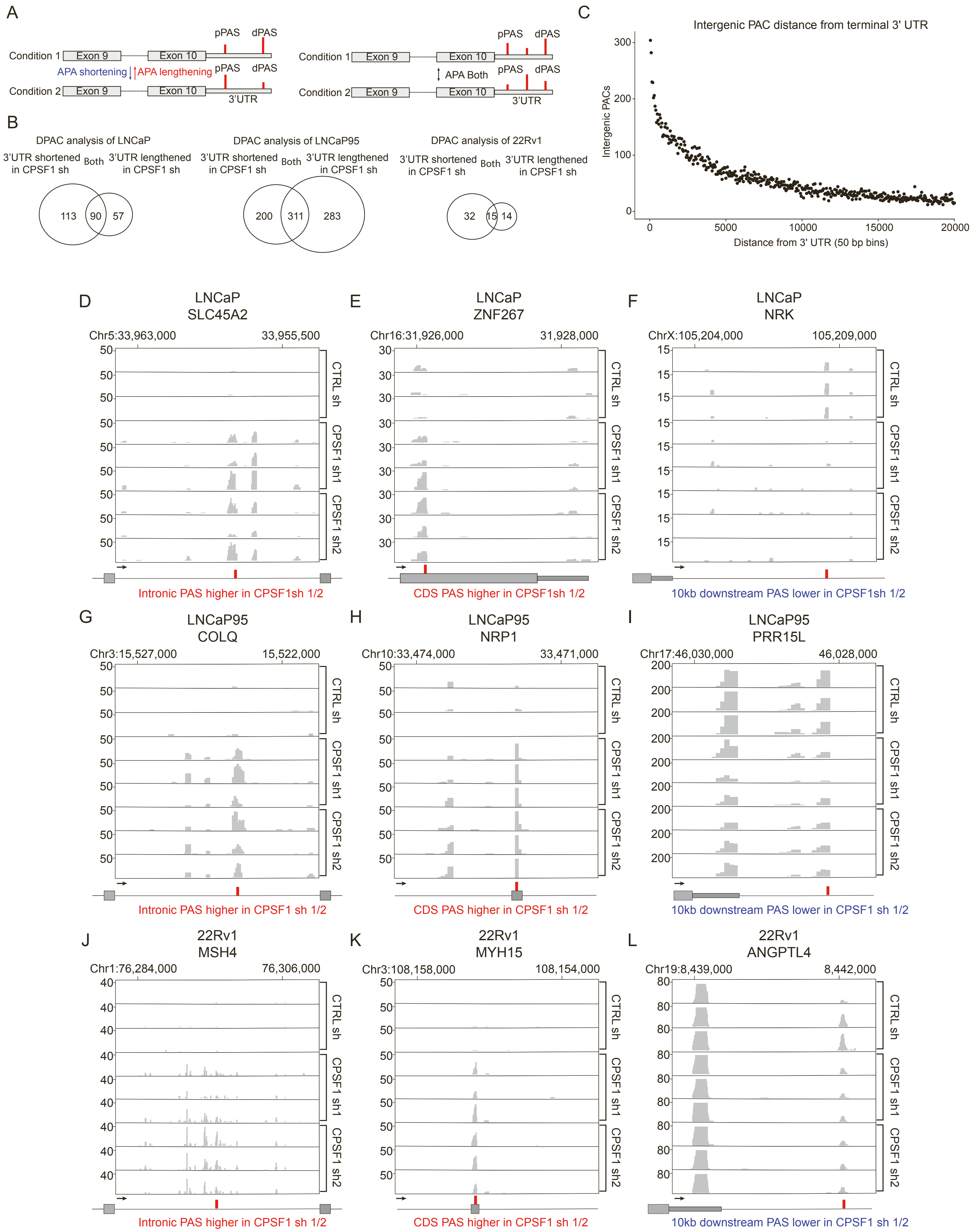

A
Condition 1
Exon 9
Exon 10
pPAS
dPAS
APA shortening
APA lengthening
Condition 2
3'UTR
APA Both
B
DPAC analysis of LNCaP
3'UTR shortened in CPSF1 sh
Both
3'UTR lengthened in CPSF1 sh
113
90
57
DPAC analysis of LNCaP95
200
311
283
DPAC analysis of 22Rv1
32
15
14
C
Intergenic PAC distance from terminal 3' UTR
Intergenic PACs
Distance from 3' UTR (50 bp bins)
D
LNCaP
SLC45A2
Chr5:33,963,000
33,955,500
CTRL sh
CPSF1 sh1
CPSF1 sh2
Intronic PAS higher in CPSF1sh 1/2
E
LNCaP
ZNF267
Chr16:31,926,000
31,928,000
CDS PAS higher in CPSF1sh 1/2
F
LNCaP
NRK
ChrX:105,204,000
105,209,000
10kb downstream PAS lower in CPSF1sh 1/2
G
LNCaP95
COLQ
Chr3:15,527,000
15,522,000
Intronic PAS higher in CPSF1 sh 1/2
H
LNCaP95
NRP1
Chr10:33,474,000
33,471,000
CDS PAS higher in CPSF1 sh 1/2
I
LNCaP95
PRR15L
Chr17:46,030,000
46,028,000
10kb downstream PAS lower in CPSF1 sh 1/2
J
22Rv1
MSH4
Chr1:76,284,000
76,306,000
Intronic PAS higher in CPSF1 sh 1/2
K
22Rv1
MYH15
Chr3:108,158,000
108,154,000
CDS PAS higher in CPSF1 sh 1/2
L
22Rv1
ANGPTL4
Chr19:8,439,000
8,442,000
10kb downstream PAS lower in CPSF1 sh 1/2

Figure S4: DPAC output for LNCaP, LNCaP95, and 22Rv1 cells with CPSF1 shRNA knockdown and examples of altered poly(A) site usage upon CPSF1 knockdown in intronic, CDS, and 10kb downstream regions of representative genes. A, Diagrams illustrating how DPAC defines 3'UTR lengthening, shortening, or a both event. **B**, Venn diagrams illustrating the number of unique genes that show either a lengthening, shortening, or both event within the annotated 3'UTR upon CPSF1 knockdown in LNCaP, LNCaP95 and 22Rv1 cells. **C**. Plot illustrating the total number of intergenic PACs detected with increasing distance from the terminal 3'UTR up to 20,000 bases in 50 base pair bins. PACs were merged from all samples across all cell lines for analysis. **D-F**, Coverage of PAC-seq reads for representative genes displaying changes in poly(A) site usage within intronic, CDS, or 10kb downstream regions upon CPSF1 knockdown in LNCaP cells. **G-I**, Coverage of PAC-seq reads for representative genes as in D-F for LNCaP95 cells. **J-L,** Coverage of PAC-seq reads for representative genes as in D-F for 22Rv1 cells. Diagrams below illustrate the effect of CPSF1 knockdown on poly(A) site usage within each indicated gene. Red lines denote poly(A) sites called by DPAC analysis. All poly(A) sites were APA = alternative polyadenylation, pPAS = proximal poly(A) site, dPAS = distal poly(A) site. bp = base pair, PAC = poly(A) cluster. CDS = coding sequence.

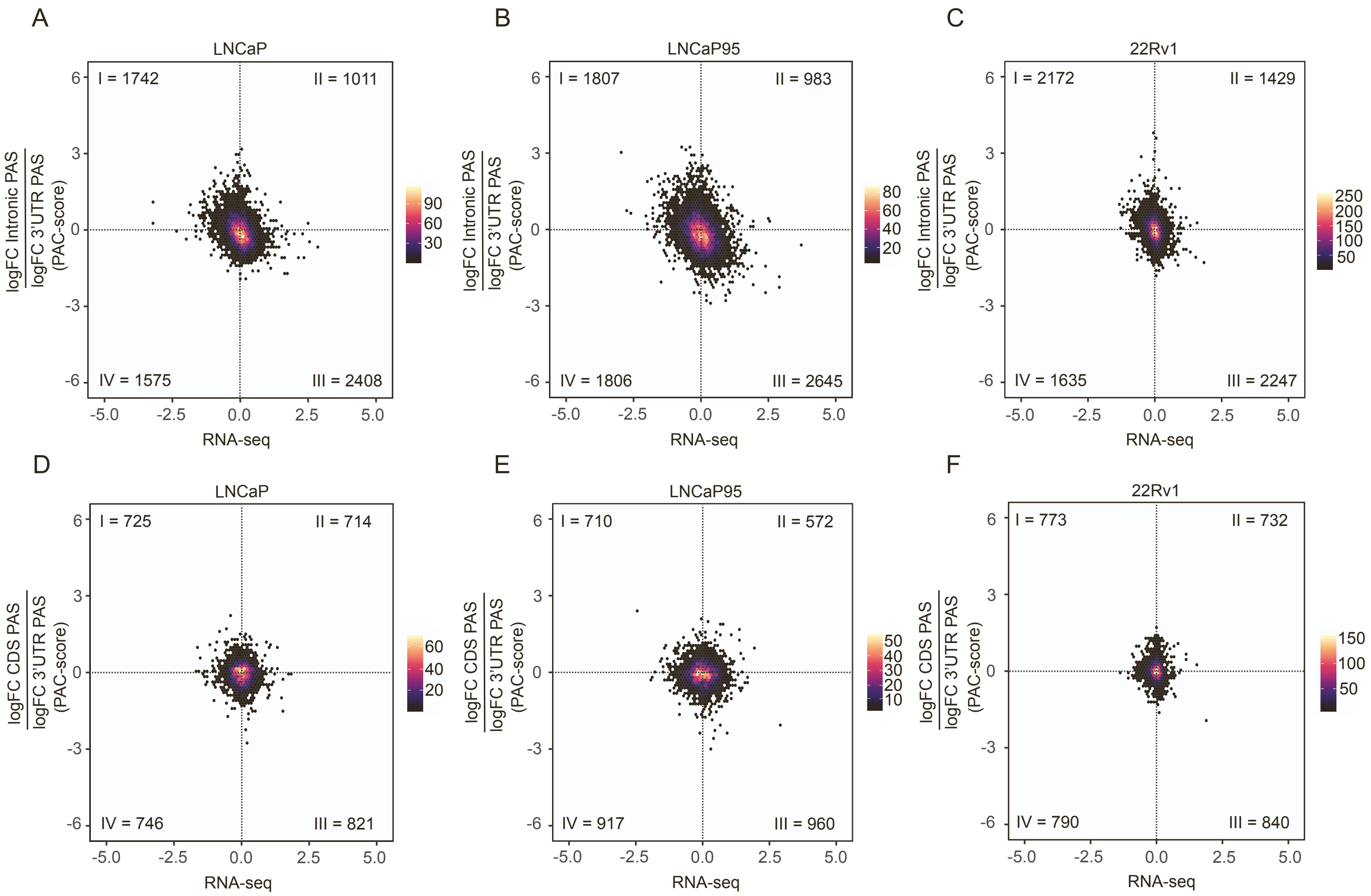
A
LNCaP
I = 1742
II = 1011
IV = 1575
III = 2408
B
LNCaP95
I = 1807
II = 983
IV = 1806
III = 2645
C
22Rv1
I = 2172
II = 1429
IV = 1635
III = 2247
logFC Intronic PAS / logFC 3'UTR PAS (PAC-score)
D
LNCaP
I = 725
II = 714
IV = 746
III = 821
E
LNCaP95
I = 710
II = 572
IV = 917
III = 960
F
22Rv1
I = 773
II = 732
IV = 790
III = 840
logFC CDS PAS / logFC 3'UTR PAS (PAC-score)
RNA-seq

Figure S5: CPSF1 regulated intronic and CDS poly(A) site usage with expression changes. A-C, Density plots illustrating the intronic PAC-score and RNA-seq differential expression of genes in LNCaP, LNCaP95, and 22Rv1 cells treated with CPSF1 shRNA. **D-F** Density plots illustrating the CDS PAC-score and RNA-seq differential expression of genes in LNCaP, LNCaP95, and 22Rv1 cells treated with CPSF1 shRNA. Numbers represent the amount of genes represented in each quadrant. CDS = coding sequence.

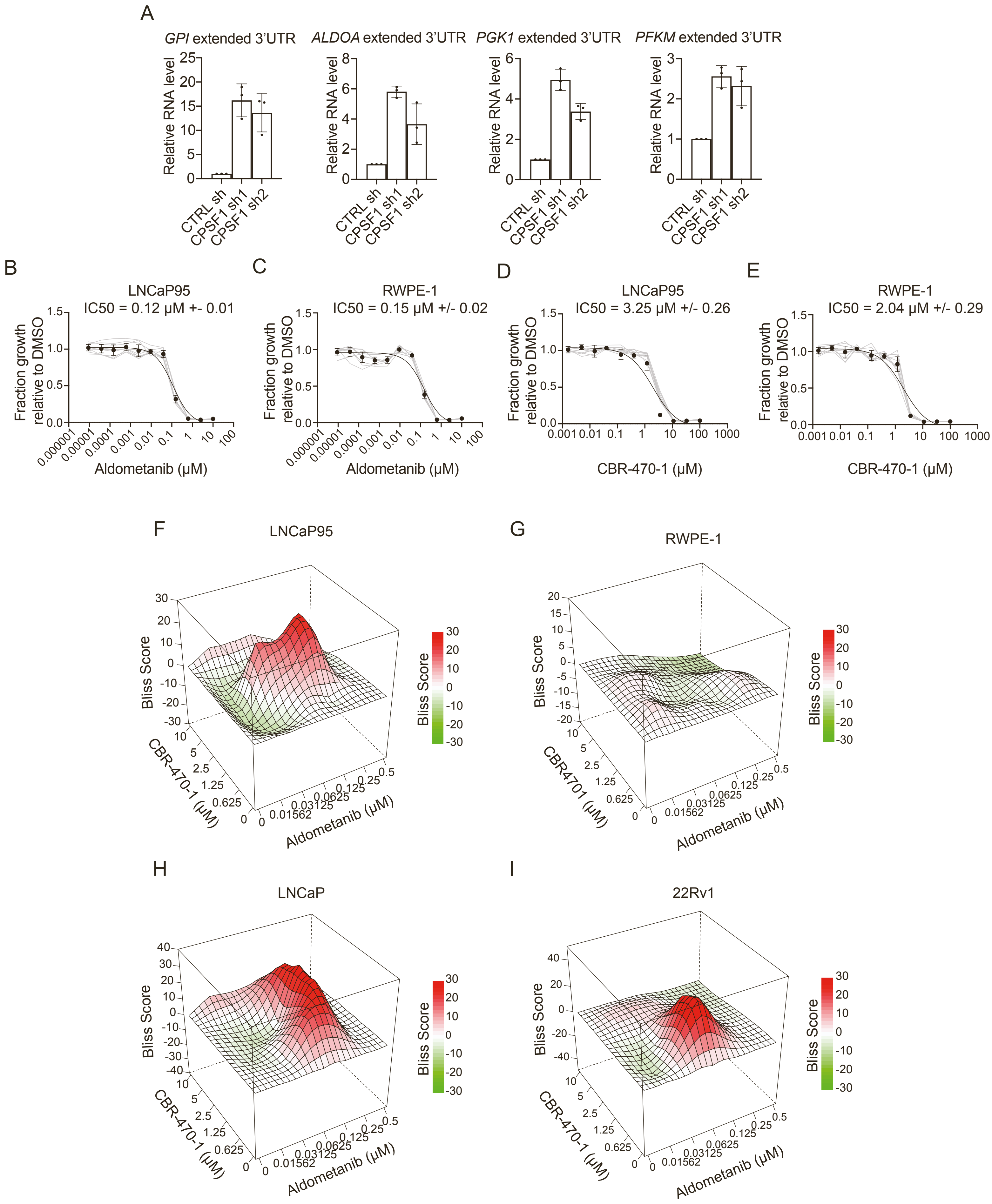

A
GPI extended 3'UTR
ALDOA extended 3'UTR
PGK1 extended 3'UTR
PFKM extended 3'UTR
Relative RNA level
CTRL sh
CPSF1 sh1
CPSF1 sh2
B
C
D
E
LNCaP95
IC50 = 0.12 μM +- 0.01
RWPE-1
IC50 = 0.15 μM +/- 0.02
LNCaP95
IC50 = 3.25 μM +/- 0.26
RWPE-1
IC50 = 2.04 μM +/- 0.29
Fraction growth relative to DMSO
Aldometanib (μM)
CBR-470-1 (μM)
F
LNCaP95
G
RWPE-1
H
LNCaP
I
22Rv1
Bliss Score
CBR-470-1 (μM)
CBR4701 (μM)
Aldometanib (μM)

Figure S6: CPSF1 knockdown results in transcriptional readthrough of genes encoding glycolysis regulators and aldometanib and CBR-470-1 display synergy for inhibiting growth of prostate cancer cells. **A**, Nascent RNA was purified from LNCaP95 cells infected with lentivirus encoding shRNAs targeting CPSF1 (CPSF1 sh1 and CPSF1 sh2) or a non-targeting shRNA (CTRL sh). RT-PCR was performed with primers targeted to the extended 3'UTR region of *GPI*, *ALDOA*, *PGK1*, and *PFKM*. **B, C**, LNCaP95 and RWPE-1 cells were treated with aldometanib at indicated concentrations and the fraction of growth relative to cells treated with DMSO (as vehicle control) was measured by crystal violet staining on day 7 after treatment was initiated. **D, E**, LNCaP95 and RWPE-1 cells were treated with CBR-470-1 at indicated concentrations and fraction growth compared to DMSO was measured on day 7 post-seeding using crystal violet staining. Gray lines indicate individual biological replicates black lines indicate the average of n = 9 biological replicates +/- 95% CI. The average IC50 of n = 9 is displayed +/- the 95% CI. **F-I**, Aggregate synergy score plots generated by the SynergyFinder program for aldometanib in combination with CBR-470-1 in LNCaP95 (**F**), RWPE-1 (**G**), LNCaP (**H**), and 22Rv1 (**I**) cells.
